# Development of a novel *S*. Typhi and Paratyphi A outer membrane vesicles based bivalent vaccine against enteric fever

**DOI:** 10.1371/journal.pone.0203631

**Published:** 2018-09-14

**Authors:** Debaki R. Howlader, Hemanta Koley, Ritam Sinha, Suhrid Maiti, Ushasi Bhaumik, Priyadarshini Mukherjee, Shanta Dutta

**Affiliations:** Division of Bacteriology, National Institute of Cholera and Enteric Diseases, Beliaghata, Kolkata, India; Universidad Nacional de la Plata, ARGENTINA

## Abstract

*Salmonella* Typhi and *Salmonella* Paratyphi A are the leading causative agents of enteric fever which cause morbidity and mortality worldwide. Currently, there is no combination vaccine which could protect infection from both the strains. In this paper, we are focusing on the development of a novel bivalent typhoidal Outer Membrane Vesicles (OMVs) based immunogen against enteric fever. We have isolated *Salmonella* Typhi and Paratyphi A OMVs and also characterized OMVs associated antigens. Then we immunized adult mice with three doses of our newly formulated bivalent immunogen orally (25 μg/200 μl). After three doses of oral immunization, we found our immunogen could significantly induce humoral response. We have also found serum IgG against LPS, Vi-polysaccharide etc. OMV immunization induces CD4, CD8 and CD19 population in immunized mice spleen. It also induces Th1 and Th17-cell mediated immunity. We also found bivalent OMVs immunization can prevent more than lethal dose of heterologous *Salmonella* strains mediated systemic infection in adult mice model. We determined that, the protective immune responses depend on the humoral and cell-mediated immune response. Furthermore, we have evaluated the mode of protective immune response carried out by anti-OMVs antibody by significantly inhibiting bacterial motility and mucin penetration ability. Taken together, these findings suggest that our bivalent immunogen could be used as a novel candidate vaccine against enteric fever.

## Introduction

Enteric fever, a serious invasive febrile illness of the human, caused by *Salmonella enterica* serovers Typhi and Paratyphi A (*S*. Typhi and *S*. Paratyphi A, respectively) is considered to be a major global burden in developed and developing countries [1]. Globally *S*.Typhi affects 21.7 million people and 200,000 deaths per year. Although *S*. Typhi is more prevalent until recently; *S*.Paratyphi (A, B and C) too can cause significant morbidity and mortality especially in Asia and in travelers returning from these endemic areas. Recent trends of enteric fever suggest a change in disease pattern, typically a rise of paratyphoid fever in the Asian and other developing countries [2, 3].

In this changing disease pattern, it has been hypothesized that, soon *S*. Paratyphi A infection would become the prime strain with significant human morbidity and mortality [2, 3]. *S*. Typhi and Paratyphi A are transmitted by ingestion of contaminated food and water. After ingestion, salmonellae adhere to the mucosa and invade intestinal epithelial cells especiallythrough Microfold (M) cells. After invasion, organisms translocate into intestinal lymphoid organs and some passes on to the liver and spleen [4]. During this stage of infection *Salmonella* activates different mucosal and systemic immune response which is very crucial for strategic suitable vaccine development.

Currently, there are three globally licensed vaccines available against *S*.Typhi, a live-attenuated oral vaccine Ty21a (*galE* mutant of the wild type Ty2 strain) and parenteral polysaccharide Vi-vaccine (Vi-vaccine) [5]. The third one is a recent tetanus toxoid-conjugated vaccine which is also against *S*.Typhi. Although the oral vaccine somewhat cross-protect paratyphoid infection, but because Vi polysaccharide is generally absent in Paratyphi, the Vi vaccine is ineffective against Paratyphi infection. Therefore, a better understanding of the immunology towards protection is required to allow more rational approach to vaccine development. In this situation, need for a new vaccine seems obvious which will protect against both *S*. Typhi and *S*. Paratyphi A infection.

Outer Membrane Vesicles (OMVs) are secreted naturally from several Gram-negative bacteria including typhoidal salmonellae. These native OMVs are being used as a next generation acellular vaccine, which induces long-term protective immune response without any additionally added adjuvant. OMVs have LPS and membrane proteins as a natural adjuvant [6, 7, 8, 9, 10, 11]. Biochemical and proteomics studies have revealed that OMVs consists of different Pathogen-Associated Molecular Patterns (PAMPs) such as LPSs, outer membrane proteins, outer membrane lipids, periplasmic proteins, DNA, RNA etc. which can activate innate as well as adaptive immune response, which is a crucial characteristic for an ideal vaccine.

The only licensed OMV-vaccine is of *Neisseria meningitides* serogroup-B vaccine, MeNZB, which is safe, immunogenic and protective in nature [12, 13]. It has showed nearly 80% protective efficacies during an epidemic meningococcal serotype B outbreak in Norway and New Zealand in both adults and children. In the past, in our laboratory, we have developed different multivalent OMV based vaccine against different enteric organisms like *V*.*cholerae*, *Shigella sp*. etc. [14, 15, 16, 17].

In this study, we are focusing onto the development of an oral bivalent OMV based typhoidal vaccine which can induce long-term broad spectrum protective efficacy against both *S*. Typhi and Paratyphi A. Another aim of our study was to prevent the entry of *Salmonella* through intestine as well as reduction in the bacterial load after systemic infection by activating mucosal as well as systemic immune response.

## Methods

### Bacterial strains and culture conditions

OMV antigens were prepared from *S*.Typhi C-6953 and *S*. Paratyphi A C-6915, and for challenge study, *S*. Typhi C-6.946 and *S*. Paratyphi A BCR 148 were collected from National Institute of Cholera and Enteric Diseases (NICED) culture bank. All strains were kept in 20% glycerol in brain heart infusion broth (Difco, USA) at −80° C. Prior to experimentation, each strain was grown in Tryptic Soy Broth (TSB; Difco, USA) at 37° C under shaking conditions (100 rpm) or on plates in Tryptic Soy Agar (TSA; Difco, NJ, USA).

### Characterization of the strains used

Strains were characterized by both PCR-based method and serotyping. For PCR, the following genes were selected and their respective primer sequences [18]have been shown in bracket:*prtA-parat-s* (5’-CTT GCT ATG GAA GAC ATA ACG AAC C-3’); *parat-as* (5’-CGT CTC CAT CAA AAG CTC CAT AGA-3’); *fliCd-as* (5’-GCA TAG CCA CCA TCA ATA ACC-3’);*fliCa-as* (5’-TAG TGC TTA ATG TAG CCG AAG G-3’); *fliCcom-s* (5’-AAT CAA CAACAA CCT GCA GCG-3’). Afterwards, anti O- and H-antibodies were used for serotypic characterization of the strains used in the study. White-Kauffmann-Le Minor scheme for Antigenic Formulae was followed during the experiment [19, 20] (S1 File).

Antibiotic-resistance pattern of the strains was also checked by Bauer method [21]. Selection of antibiotics was dependent on both WHO, CLSI guidelines [22, 23] and the availability of antibiotics in our facility. Antibiotic disks (AMP 10 μg, CTX 30μg, CFZ 30 μg, CFX 5μg, CRO 30 μg, CHL 30 μg, I 10 μg, NAL 30 μg, CO 25 μg, TET 30 μg, TMP 30 μg, SXT 23.75 μg, LO 30 μg, AZ 15 μg, CIP 5 μg, STR 10 μg, KAN 30 μg) were purchased from BD Bioscience, USA (S2 File).

### Preparation of OMVs

OMVs were prepared from two *Salmonella enterica* strains as shown in Table 1 following the method adapted by Sinha et al. with slight modifications [15]. Briefly, cells were grown till exponential phase at 37°C under shaking condition followed by centrifugation at 8000 rpm for a total of 40 minutes at 4°C. Following filtration by 0.22 μm bacterial filters (Millipore, USA), OMVs were subsequently purified by ultra-centrifugation (4 h, 140,000 x g, 4°C) using a Sorvall T-865 rotor, and re-suspended in Phosphate-Buffered Saline (PBS, pH 7.4). Protein concentration was determined by the modified Lowry protein assay kit (Pierce, USA). Reducing sugar in LPS O-Ag was determined by a method used by Dubois et al [24].

**Table 1:** Concentration of protein and O-antigen in OMV samples.

*Name of OMVs*	*Mean concentration of protein (μg/ml)*	*Mean concentration of O-antigen (μg/ml)*	*Source or reference*
*S*. Typhi C-6953	414	250	This study
*S*.Paratyphi A C-6915	430	235	This study

The amount of protein and O-antigen of LPS associated with isolated OMVs were measured by Lowry and Dubois methods, respectively. The amount of protein varied between 400–440 μg/ml in every batch. The exact amount was calculated every time before the introduction of the immunogen in animals or in *in vitro* study.

### Negative staining of OMVs and OMV-secreting bacteria

A 5 μl aliquot of secreted OMVs were placed on a carbon coated grid and left for 1 minute for proper absorption. The grid was then washed with two drops of Tris-HCl buffer. After blotting excess fluid, the sample was stained with 2% aqueous solution of uranyl acetate. In case of negative staining of bacteria, the same procedure was followed with log-phase live bacterial cells. Both the negatively stained OMVs and bacteria-secreting OMVs were observed under Tecnai 12 (Bio Twin Transmission Electron Microscope, FEI, the Netherlands) operating at 80 kV (Hayat & Miller, 1990).

### MALDI-TOF/TOF

Standard procedure was followed for the preparation of samples for MALDI-TOF/TOF [25]. Coomassie stained SDS-PAGE gel pieces were excised. Gel pieces were processed using in-gel tryptic digestion kit (Pierce, Thermo scientific). In brief, they were reduced with Tris (2-carboxyethyl) phosphine in ammonium bicarbonate buffer (25 mM), incubated with iodo-acetamide in dark for 1 h and subsequently digested overnight with trypsin (100 ng/ sample) in ammonium bicarbonate buffer.

Gel pieces were washed with acetonitrile, dried and further rehydrated with trypsin containing digestion buffer. Peptides were extracted from gel, dried, and dissolved in acetonitrile (50%) in trifluoroacetic acid (0.1%). Subsequently, they were spotted on a target MALDI plate using Cyano Hydroxy Cinnamic Acid (CHCA) as a matrix and analyzed using MALDI-TOF mass-spectrometer (Applied Biosystem, Foster City, CA). Spectra were calibrated using the matrix and tryptic auto-digestion ion peaks of Calmix, a standard mixture of six peptides.

Spectral data were analyzed from PMF in combination with MS/MS spectra by searching against the database using the MASCOT (Matrix Science Ltd., London, UK) version 2.2 and basic local alignment search tool (BLAST) of ABI GPS Explorer software, version 3.6 (Applied Biosystems). For database searching the following parameters were used. Peak list-generating software: 4000 series explorer software version 3.5; taxonomy: all entries; database: MSDB version 2.1.0 dated 27.02.2005; No of entries: Database-MSDB20050227 (1942918 sequences; 629040812 residues); cleavage enzyme: trypsin; variable modifications: oxidation on methionine; fixed modification: carbamidomethylation; missed cleavages permitted: one missed cleavages; minimum signal to noise ratio (S/N): 10; peptide charge: +1; precursor mass tolerance: +/- 100 ppm; mass tolerance for the MS/MS search: +/- 0.2 Da. Significance of data was selected according to their *p* value (*p* < 0.05) where p is the probability that the observed match in a random event. Therefore, Mascot search engine is setting the threshold ions score [-10*Log(p)] on its own based on the type of analysis, number of spectra to be analyzed etc.

### Animals

Seven weeks old female BALB/c mice of either sex were taken from the animal resource division of NICED, Kolkata. Female mice were caged separately as groups of 10 and maintained at a temperature of 25°C with humidity at 75%. Mice were fed sterile food and water. All the animal experiments were conducted following the standard operating procedure as outlined by Committee for the Purpose of Control and Supervision of Experiments on Animal (CPCSEA), Ministry of environment and forest, Government of India. The animal experimental protocol was approved by the Institutional Animal Ethical Committee of NICED with the project approval no. PRO/117/June, 2015–June, 2018.

### Oral immunization

Seven weeks old female BALB/c mice were kept empty stomach for (3–4 h) hours before the immunization date, water *ad libitum*. Mice were immunized orally on day 0^th^, 14^th^ and 28^th^ with 25 μg of purified *S*. Typhi, or *S*. Paratyphi A OMV and a combination of both the OMVs in a 1:1 formulation in 200 μL of PBS following the protocol as explained previously as shown (S3 File).

### Collection of serum and stool

Blood was collected from the lateral tail vein at different time intervals on the 0^th^, 14^th^, 21^st^, 28^th^, 35^th^, 78^th^ and 90^th^day of first oral immunizationas shown in S3 File. The collected blood was taken in BD Microtainer (BD, NJ, USA) followed by centrifugation (1000 rpm, 10 min and 4°C).

Stools were collected aseptically from immunized and non-immunized mice at the same time intervals as blood. Stools were then homogenized in protease inhibitor (MP Biomedicals, USA)-containing PBSand centrifuged at 10000 x g for 10 min to remove the debris. The supernatant was collected and stored at -20°C for further use.

### Isolation of outer membrane proteins (OMPs) of S. Typhi and Paratyphi A

Isolation of OMP was carried out as stated elsewhere with modifications [26]. For isolation of outer membrane proteins of two *Salmonella* strains, 50 ml of the respective strains were grown till late exponential phase. Cells were harvested (4,500 x *g*, 15 min, 4°C), washed once and then re-suspended in 10 mM HEPES, pH 7.5, with protease inhibitor (MP Biomedicals) and then sonicated in a Hielscher (UP100H) ultrasonic processor. Unbroken cells were removed by centrifugation (13,000 x *g*, 1 min). The supernatant containing the OM proteins was transferred into a new tube and centrifuged again (13,000 x *g*, 30 min, 4°C). The pellet was re-suspended in 0.8 ml of 10mM HEPES, pH 7.5, plus 1% sarcosyl (Sigma) and incubated for 30 min. After centrifugation (13,000 x *g*, 30 min, 4°C), the pellet was washed once with 1 ml of 10 mM HEPES, pH 7.5, and re-suspended in 50 μl of 10 mM HEPES, pH 7.5. The protein concentration was determined as described above and adjusted to 2.5 μg/μl by using 10 mM HEPES, pH 7.5. Purified OM proteins were stored at -80°C for further use.

### Isolation of LPS

LPS was isolated by the protocol followed previously [27]. Briefly, overnight cultures of two strains were centrifuged followed by washing with PBS. Phenol-saturated 3-[N-morpholino] propen sulfonic acid (MOPS) was added and centrifuged at 15000 x g for 20 minutes. Four volume of chilled ethanol was then added and kept at -20°C overnight. Next day, the LPS was isolated and dissolved in autoclaved, distilled water and kept at -20°C for further use.

### TCA precipitation

Culture supernatant was taken out and TCA was used to precipitate the proteins dissolved in it as described elsewhere [28].

### Isolation of whole cell lysate (WCL)

Bacteria were grown overnight and centrifuged at 8000 rpm for 10 minutes, washed with PBS (Sigma, USA, pH 7.4) and sonicated in a Heilcher UP100H sonicator at 100% amplitude/cycle for at least 12 cycles of 30 seconds each. Cells were then checked for membrane lysis and centrifuged at 10,000 rpm for 10 minutes. The supernatant was collected and stored at -80°C for future use.

### SDS-PAGE and immunoblot

Total protein content of the OMVs along with OMV-associated LPS profile in the isolated OMVs, mature LPS and OMPs from *Salmonella* strains were determined by SDS-PAGE. OMVs were incubated with Proteinase-K and SDS at 60°C for 2 hours to isolate the OMV-associated LPS.80 μg of OMVs, OMPs, were boiled in SDS-PAGE buffer and all four LPS samples were boiled in LPS sample buffer. The samples were then loaded onto a 12% SDS-PAGE gel separately depending on their staining reagent. 100 V was then applied for running the gel in an AE-6530 SDS-PAGE apparatus from ATTO Corporation (Japan). The gel was then stained by either coomassie or silver stain.

Afterwards, we did immunoblot assay to determine immunogeniccomponents present in the same samples along with WCL. The samples were electrophoresed together, transferred ontonitrocellulose membrane (Bio Rad, USA) by using ATTO Corporation AE 6687 (Japan) semi-dry blot apparatus for Western blot analysis. Anti-OMV serum from immunized mice was used. Presence of immunogenic components in OMVs was further determined by using convalescent serum from mice. Here, only OMVs were electrophoresed and blotted using convalescent serum. Blots were either observed by using alkaline phosphatase substrate BCIP/NBT (MP, USA, Cat# 980621) or Amersham^™^ ECL^™^ Prime Western Blotting Detection Reagent (GE Healthcare, UK, Lot 15839044). In all the cases, same condition and equipment were being followed and used, respectively.

### Dot blot assay

Dot blot analysis was done as described previously [27]. Briefly, LPS from two strains were taken and blotted directly onto a nitrocellulose membrane in different concentrations. The membrane was then washed with Tris-Buffered Saline (TBS) containing 0.1% Tween-20. The membrane was then incubated with anti-OMV anti-sera from immunized mice and anti-mouse IgG-HRP secondary antibody (Sigma, USA) successively and the blot was then finally developed by chemiluminescence.

### ELISA

Different immunoglobulins; e.g. IgG and its sub-types (IgG1, IgG2a, IgG3), and IgA, sIgA and IgM were measured by ELISA as stated by Keren [29]. Briefly, disposable polystyrene micro-titer wells (Nunc, Denmark) were separately coated with two different typhoidal OMVs (5 μg/well) as shown in Table 1 and incubated for 18 h at 4°C. Wells were washed and blocked with Bovine Serum Albumin (BSA; Sigma-Aldrich, USA). After washing the wells with PBS-T (PBS with 0.5% Tween-20, Sigma-Aldrich, USA) and incubated with serially diluted serum samples, 100μL HRP-conjugated goat anti-mouse immunoglobulin was added and incubated. After washing with PBS, the substrate o-phenyl-Di-amine (OPD) was added to each well followed by stopping the reaction after 10 min by adding 100 μL of 2 N sulphuric acid. OD_492_ was taken afterwards. The experiments were repeated three times for each immunoglobulin, with the immunized and non-immunized serum, collected from individual mice, before, during and after immunization. The same procedure was carried out when ELISA were done against Vi-polysaccharide (Batch number: T030516, Bio-Med, India) of *S*. Typhi.

### Measurements of CD4+, CD8+ and CD19 expression in splenic cells by flow cytometry analysis

Spleens were collected from both naïve and immunized BALB/c mice and stained for FACS analysis. Expression of CD4, CD8a and CD19 were checked by using PE-tagged antibodies from Miltenyi Biotech, Germany (CD4-PE: 130-116-509; CD8a-PE: 130-109-247; CD19-PE: 130-12-035, REA control: 130-104-613) [30]. Expression was checked using a FACS Aria II flow cytometry machine. Data was being calculated using a FACSDiva version 6.1.3 software. Percent up-regulation was measured using the following formula:

%up-regulation=Deltameanfluorescenceintensity/deltameanPE-Ax100;

Where delta mean fluorescence intensity was the difference between delta mean fluorescence intensity of immunized–delta mean fluorescence intensity of control.

### Cytokine assays

#### i. Splenocyte re-stimulation assay

After 1 week from the end of last immunization, splenic cells from immunized BALB/c mice were cultured for 2 hours in complete RPMI 1640 (MP Biomedicals, USA) containing 10% FBS (Gibco, Life Technologies, USA). Cells were then treated with 10 μg/ml of bivalent OMV and incubated in 37°C for 24 hours in presence of 5% CO_2_ [15]. Different cytokines, namely IFN-γ, IL-6 and IL-17 were then measured by cytokine ELISA kit (Invitrogen, USA).

#### ii. Isolation of bone marrow-derived dendritic cells (BMDCs)

Immature bone marrow was isolated from naïve BALB/c mice and kept in RPMI 1640 medium supplemented with 10% FBS, Granulocyte-Macrophage Colony Stimulating Factor (GM-CSF) until their maturation. Matured BMDCs were stimulated with 10 μg/ml of bivalent OMV for 24 hours in presence of 5% CO_2_ [15]. Th17-polarizing cytokines were then measured using cytokine ELISA kit (Invitrogen, USA).

#### iii. BMDC-splenic CD4 T cell co-culture

BMDCs were isolated from naïve BALB/c mice and cultured like mentioned above. Splenic cells from both naïve and immunized BALB/c mice were collected, CD4 T cells were isolated (Dynal^®^ Mouse CD4 Negative Isolation Kit, Cat. No. 114.15D, Invitrogen Dynal AS, Oslo, Norway) and cultured with BMDCs at a 4:1 ratio for 8 hours in an U-bottom microtiter plate under the same condition with a stimulation of 10 μg/ml bivalent OMV [31]. Th1 and Th17-polarizing cytokines were measured using cytokine ELISA kits from Invitrogen, USA according to the manufacturer’s instructions.

### Intra-peritoneal mice model for typhoidal and paratyphoidal salmonellae and determination of LD_50_

Mice were challenged intra-peritoneally with 1 x 10^3^ to1 x 10^9^ doses of challenge bacteria for the determination of LD_50_ value. A total of 24 mice were divided into four groups. 6 mice per group were challenged intra-peritoneallywith one of the following four bacterial concentrations: 1 x 10^3^ CFU/ml, 1 x 10^5^ CFU/ml, 1 x 10^7^ CFU/ml and 1 x 10^9^ CFU/ml. The mice were then kept for 7 days to record mortality based on humane endpoint analysis. In all cases, including survival analyses, the following points were considered while determining the humane endpoint: reduction in body weight (more than equal to 20% than that of the healthy animals), with severe lethargy, reduction in rectal temperature (more than equal to 3 degree C than that of the healthy animal). Mice were then kept for 4 more hours to observe whether their health situation can revert back to normal. After the elapse period, they were euthanized using Ketamin hydrochloride via parenteral route. This preliminary study on the mortality of mice indicated the LD_50_ value must lie somewhere between 1 x 10^7^ CFU/ml and 1 x 10^9^ CFU/ml. So, we then challenged a group of 12 mice (which were divided into 2 separate groups; i.e. 6 in each group) fresh mice with the following concentrations of bacteria: 2 x 10^7^ CFU/ml and 2 x 10^8^ CFU/ml. After finding the LD_50_ value to be 2 x 10^7^ CFU/ml for both the challenge strains, mice were infected intra-peritoneally [32] with LD_50_ dose to check the systemic spread of the bacteria. They were kept in normal condition in the animal cages. The infected mice were also observed for physiological changes in their body such as hunched back, lethargy, piloerection etc. Internal organs like spleen and liver were isolated on 1 to 5 dayspost infection (1–5 dpi) for the detection of bacteria in the organs. Colonization in small, large intestine and stool were also evaluated. After the proper repetition and development of this mice model, we moved forward towards functional protective efficacy studies. In all the cases, remaining mice were euthanized at the end of the experiment. Mice were checked frequently at a 1-hour interval.

### Protective efficacy studies

#### i. Challenge study

In challenge study, we have taken three groups of mice, 10 animals per group. The animals of each non-immunized, immunized and PBS group were challenged intra-peritoneally with (>LD_50_) 1 x 10^8^ CFU/ml of heterologous virulent bacteria after one week of last immunization. After challenge, the infected mice were kept for 9 days for monitoring and survival and colonization studies. There was one more group, namely the PBS-challenged group which was inoculated with 100 μl PBS and kept for 9 days. The same system was followed for mice when they were challenged with sub-lethal dose (3 x 10^5^ CFU/ml) heterologous typhoidal strains. Spleen and liver were isolated, homogenized, serially diluted and plated to see the bacterial content of the infected animals 3 Days Post Infection (DPI). Bacterial load in small intestine was evaluated on 3–5 DPI from 3 mice per group.

#### ii. Adoptive transfer

Serum and spleen from immunized and non-immunized mice were obtained 1 week after the last immunization. For serum transfer, each naïve mouse was injected via tail vein with 100 μl from either OMV-immunized or non-immunized mice. For splenocyte transfer, spleens were disassociated with a cell strainer and a sterile plunger from a 10-ml syringe. After the R.B.C.s were lysed by R.B.C. lysis solution (Sigma-Aldrich, USA), splenocytes were re-suspended in PBS and 100 μl of this cell suspension (containing 1 x 10^6^ splenocytes) was injected in naïve mice via tail vein. Mice were then challenged intra-peritoneally with heterologous strains of the same species at a concentration of 1 x 10^6^ CFU/ml to observe the passive protection, if any. One group of mice was challenged on the same day of the adoptive transfer and the other group was challenged after seven days. Spleen and liver were isolated on 3^rd^ day post-infection, homogenized, serially diluted and plated as indicated before.

### Serum agglutination

Challenge strains were transformed with GFP plasmid. 1 x 10^8^ CFU/ml log-phase GFP-tagged challenge cells were incubated with heat-inactivated non-immunized and immunized sera at 37°C for 1 hour for classical serum agglutination to occur. Visible clumps were then observed under fluorescence microscope.

### Motility assay

Motility assay was done as previously described, with modifications [33]. Briefly, heat-inactivated immunized and non-immunized serum samples were mixed with PBS at a concentration of 1:400 and poured on soft agar (0.3%) plates. The plates were kept for an hour to get the serum mixed PBS to soak in the plate. After the plates became dry, log-phase bacteria (OD_600_ = 0.8) were pricked in the middle of the plate. The plates were then incubated at 37°C for 24 hours.

### Mucin penetration assay

The assay was performed according to previously described protocol [34]. In brief, 1% mucin columns were prepared in1 ml syringes. Bacteria were incubated with heat-inactivated non-immunized and immunized sera for 1 hour at 37°C. Cultures were then taken and 100 μl of culture containing equal number bacteria (1 x 10^7^ CFU/ml) were added from the top of 1% mucin columns. Columns were then kept at 37° C under static conditions. After 30 min of incubation 500 μl fractions were collected from the bottom of the columns, serially diluted and plated onto TSA to measure the bacterial count.

### Opsonization assay

Opsonization by peritoneal macrophage was done as described previously [35]. Briefly, mouse peritoneal macrophages were harvested by flushing the peritoneal cavity of BALB/c mice with ice-cold PBS. Collected cells were then centrifuged and re-suspended in pre-warmed RPMI (Gibco, USA) media supplemented with 10% FBS. Approximately, 5 x 10^5^cells were seeded to each well in 12-well plates and cultivated in CO_2_-incubator (Thermo Fisher Scientific, USA) for 2 hours at 37°C. Cells were washed and then log-phase bacteria (either immune serum-treated or non-immune serum-treated) were added at a M.O.I. of 1:100 and were kept for 30 minutes. Cells were washed with PBS and incubated with pre-warmed RPMI media for another 30 minutes. Then the cells were washed and incubated in pre-warmed RPMI media for 0 and 60 minutes. Finally, the cells were lysed with 0.1% Triton X-100 and serially diluted and plated.

### Statistical analysis

In the majority of cases, the data presented are not normally distributed due to biological variation. Therefore, non-parametric tests were used for all data analysis. Comparison between two categorical variables was made using the two-tailed student’s t test. Comparison between multiple categorical variables was made using the one-tailed student’s t test. Each experiment was repeated at least three times. A *P* value of either <0.05 or <0.01 were considered significant. GraphPad Prism 5 and Microsoft Excel, both were used in Windows OS for all the statistical analyses.

## Results

### Basic characterization of salmonella strains used in the study

As our strains were clinical isolates, we wanted to be assured about their genotypic and phenotypic properties. Presence of genus-specific *prtA* in all four strains and presence of *fliC-j* in *S*. Typhi strains and *fliC-a* in *S*. Paratyphi A strains were observed. Presence of FliC-j was determined by PCR-based method, whereas only FliC-d was observed via serological studies. This has been reported previously and perfectly coincides with a previous data and supports the genotypic evidence that our strains were indeed typhoidal salmonellae as shown in S1 File [18, 36, 37]. Very small change in gene level may sometimes be unrecognizable in serological motility assays, which increases the chance of a false result (S1 File) [35, 38]. But since our objective was just to prove that the strains are indeed either *S*. Typhi or *S*. Paratyphi A, we have not done further classifications.

### Isolation and characterization of S. Typhi and S. Paratyphi A OMVs

**Fig 1:**
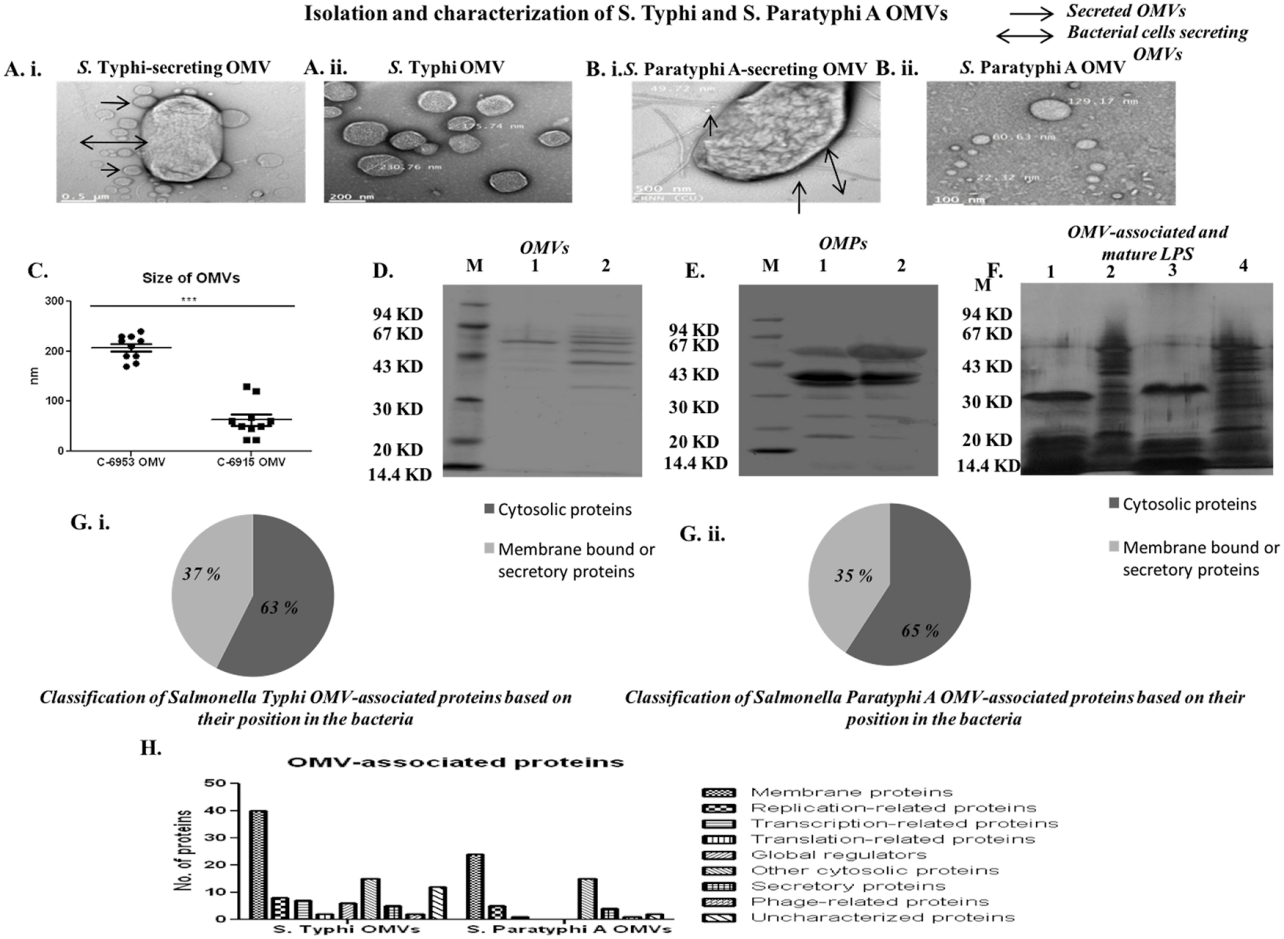
Isolation and characterization of OMVs. **A. i**. OMVs attached to *S*. Typhi bacteria and **A. ii**. Isolated OMVs from *S*. Typhi. **B. i**. OMVs attached to *S*. Paratyphi A and **B. ii**. Isolated OMVs from *S*. Paratyphi A. **C**. Sizes of isolated OMVs.10 different OMVs were chosen from 10 different fields and their sizes were noted down. Each dot represents an individual OMVs size. **D.**SDS-PAGE profile of OMVs extracted from two strains of typhoidal salmonellae. Lane M: Low molecular weight marker (Bangalore GeNei), Lane 1: *S*. Typhi, Lane 2: *S*. Paratyphi A OMVs. **E.**SDS-PAGE profile of OMPs extracted from two strains of typhoidal salmonellae. Lane M: Low molecular weight marker (Bangalore GeNei), Lane 1: *S*. Typhi, Lane 2: *S*. Paratyphi A OMPs. **F.** Different natures of OMV-associated and mature LPS. Lanes 1 & 3: Proteinase K-treated OMV of *S*. Typhi and *S*. Paratyphi A, respectively. Lanes 2 & 4: Extracted mature LPS of *S*. Typhi and *S*. Paratyphi A, respectively. **G. i.** Pie-chart showing the presence of hypothetical cytosolic and membrane-bound proteins in *S*. Typhi OMVs. **G. ii.** Pie-chart showing the presence of hypothetical cytosolic and membrane-bound proteins in *S*. Paratyphi A OMVs. Both cases indicate the abundance of cytosolic proteins instead of membrane-bound proteins for both OMVs. **H.** Classification of OMV-associated hypothetical proteins according to their functions. The OMV-associated proteins unique to *S*. Typhi and Paratyphi A were grouped based on their biological processes and molecular functions, and their proportions are plotted.

Both *S*. Typhi and Paratyphi A secrete OMVs during log phase of growth (Fig 1Ai and 1Bi). In electron microscopic analysis, we found different size of isolated OMVs. The sizes of these OMV’s were distributed between 130–300 nm (Fig 1Aii and 1Bii) and the *S*. Typhi OMVs were relatively larger in size in comparison to the *S*. Paratyphi A (Fig 1C). Then, we quantified the protein and O antigen of both OMVs and we found both OMVs contained nearly 400 μg/ml protein and 250 μg/ml O-Ag as shown in Table 1 above. Furthermore, SDS-PAGE analysis showed protein bands of 40KD to 62KD in case of *S*. Typhi OMVs, whereas, bands of 33KD to 73KD were shown in case of *S*. Paratyphi A OMVs. Other than that, faint bands were observed in both cases (Fig 1D). We further evaluated the native OMPs of these two typhoidal salmonellae to compare the protein profile with isolated OMVs. A vast difference in banding patterns (Fig 1E) indicated that our OMVs contain constituents other than the OMPs.

We further evaluated the OMV-associated LPS after chemically neutralizing the proteins with Proteinase-K. Proteinase-K treated OMVs were found to be associated with LPS of low molecular weight which might be an effect of the change in the structure of OMVs after the treatment (Fig 1F). Lanes 1 and 3 in Fig 1F are OMV-associated LPS isolated from the OMVs. On the other hand, lanes 2 and 4 in the same figure are native, mature LPS isolated from the bacteria itself. The bands located between the core and lipid A represent LPS synthesis intermediates consisting of the core and different amounts of oligosaccharide subunits in case of native LPS preparation [39]. Literature suggests, the native LPS isolated from the bacteria itself contains O-Ag, core polysaccharide and lipid A. On the other hand, the OMV-associated LPS contain mature LPS structure and free lipid A. In case of *S*. Typhimurium, OMVs contain deacylated LPS, but the bacteria have hexa-acylated LPS [40]. This might be another reason of the observed change in banding pattern. This also indicates the vesicles not remaining intact once the treatment was over. This might also be the reason of the structural dissimilarity observed. As the banding pattern was much different in TCA precipitation, our immunogen must contain OMVs as a major constituent (S12 File).

### Determination of the OMV proteome

The MALDI-TOF/TOF spectrums were shown in S4 and S5 Files. A total of 100 hypothetical proteins in case of *S*. Typhi OMVs as shown S6–S9 Files and 42 hypothetical proteins in case of *S*. Paratyphi A OMVs as shown in S10 and S11 Files were found. The comparison of the sub-cellular locations of the OMV protein components with that of *Salmonella* genome-inferred proteins revealed that OMVs derived in the exponential phase of growth cycle contains both cytosolic as well as membrane proteins (Fig 1Gi and 1Gii). The proteomic profiling of these OMVs (Fig 1H) revealed hypothetical protein compositions in the isolated OMVs, which indicates that the OMVs cargo depends on the growth conditions and provides a deeper insight into how *Salmonella* utilizes OMVs to adapt to environmental changes. Although the proteomic analysis does not suggest that these proteins are the sole proteins that are present in OMVs, but these are the proteins which occur most abundantly in OMVs. There might be a number of other proteins which we were unable to isolate using SDS-PAGE.

### Immunogenicity of bivalent OMVs

#### i. Activation of humoral immune response by bivalent OMVs

**Fig 2:**
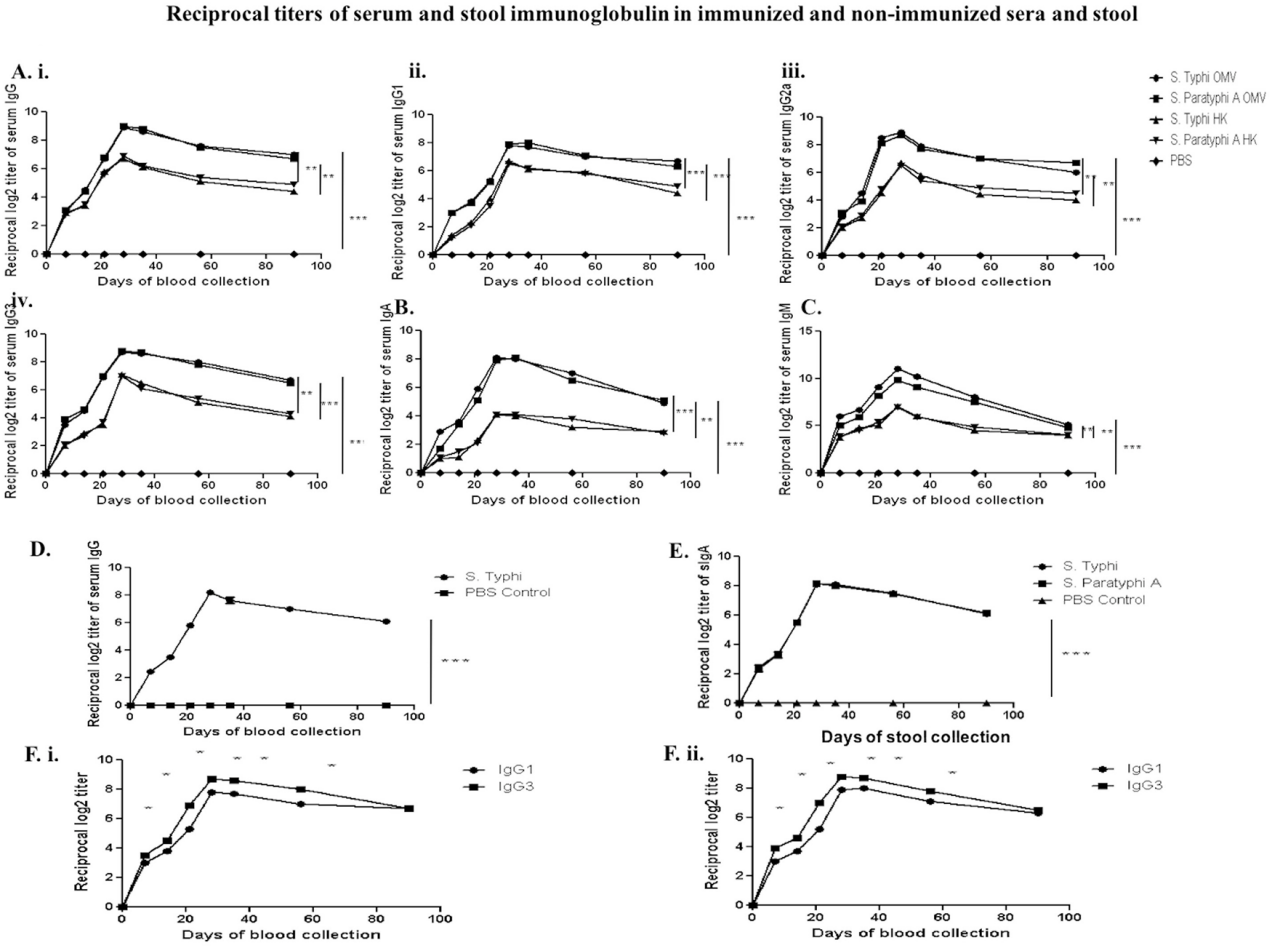
Serum immunoglobulin titers in immunized sera were separately measured against each component OMVs of bivalent OMV and heat-killed (HK) formulations. ELISA plates were coated with either each component of OMVs or heat-killed immunization and either bivalent immunized mice serum or heat-killed immunized mice serum was used, at appropriate times. While doing ELISA against Vi-polysaccharide, ELISA plates were coated with market-available Vi-polysaccharide. **A.** Serum IgG (i), IgG1 (ii), IgG2a (iii), IgG3 (iv); **B.** Serum IgA; **C.** Serum IgM; **D.** Anti-Vi serum IgG; **E.** sIgA; **F.** i. ii. Serum IgG1 and IgG3 response against each of the two OMVs, *Salmonella* Typhi and Paratyphi A, respectively at pre-immunization, immunization and post-immunization periods. High serum IgG3 titer against IgG1 indicates higher Th1 cell-mediated immune response in adult mice sera after three doses of immunization. The horizontal axis indicates the days of blood collection. Data represented here are the mean values +/- Standard Deviation (SD) of three independent experiments. No statistically significant difference in overall antibody response was observed against each of the two OMVs (P values >0.05). The differences in post-immunization day wise response of each of the studied antibodies against each of the two OMVs were highly significant (P value< 0.005).

In the next experiment, we measured the generation of systemic as well as mucosal humoral immune response after oral immunization of female mice with four doses (25 μg/dose) of bivalent immunogen. Our data showed significantly higher levels of serum IgG, IgG1, IgG2a, IgG3, IgM, IgA antibodies against each OMV up to 90-days post 1^st^ immunization. The responses differed from each other significantly (P value < 0.005). Significant higher level of IgG1, IgG2a, IgA were seen after the second dose of immunization. Although the titers were found to be above the detection level even after 90-days from day 0, the highest peaks were found to be between day 28 and day 35. Simultaneously, we compared immune responses between OMVs and heat-killed immunogen and we found bivalent OMVs are more immunogenic than heat-killed immunogen. (Fig 2A, 2B and 2C).

*S*. Typhi has a capsular Vi-polysaccharide on the outer membrane [41]. In our study, we have found that adequate amount of Vi-polysaccharide specific anti-Vi-polysaccharide serum IgG was generated after oral immunization (Fig 2D). We also observed mucosal immune response from stool diluents and found the same trend as noted before in case of serum antibodies (Fig 2E).

Moreover, our bivalent typhoidal salmonellae OMV formulation induces a higher IgG3 response than IgG1, against each component of OMVs (Fig 2Fi and 2Fii). This result indicates that, bivalent OMVs immunization elicited Th1-cell mediated immunity, manifested by higher IgG3 response than IgG1 against each component of the OMV. The overall antibody titer was increased during the immunization process and it reached a peak near 28-day post-immunization. The immunoglobulins were detectable until day 90. From, the above result we can conclude that our bivalent immunogen is immunogenic and more immunogenic than heat-killed immunogen.

#### ii. Specificity of the antibody response via immunoblot

**Fig 3:**
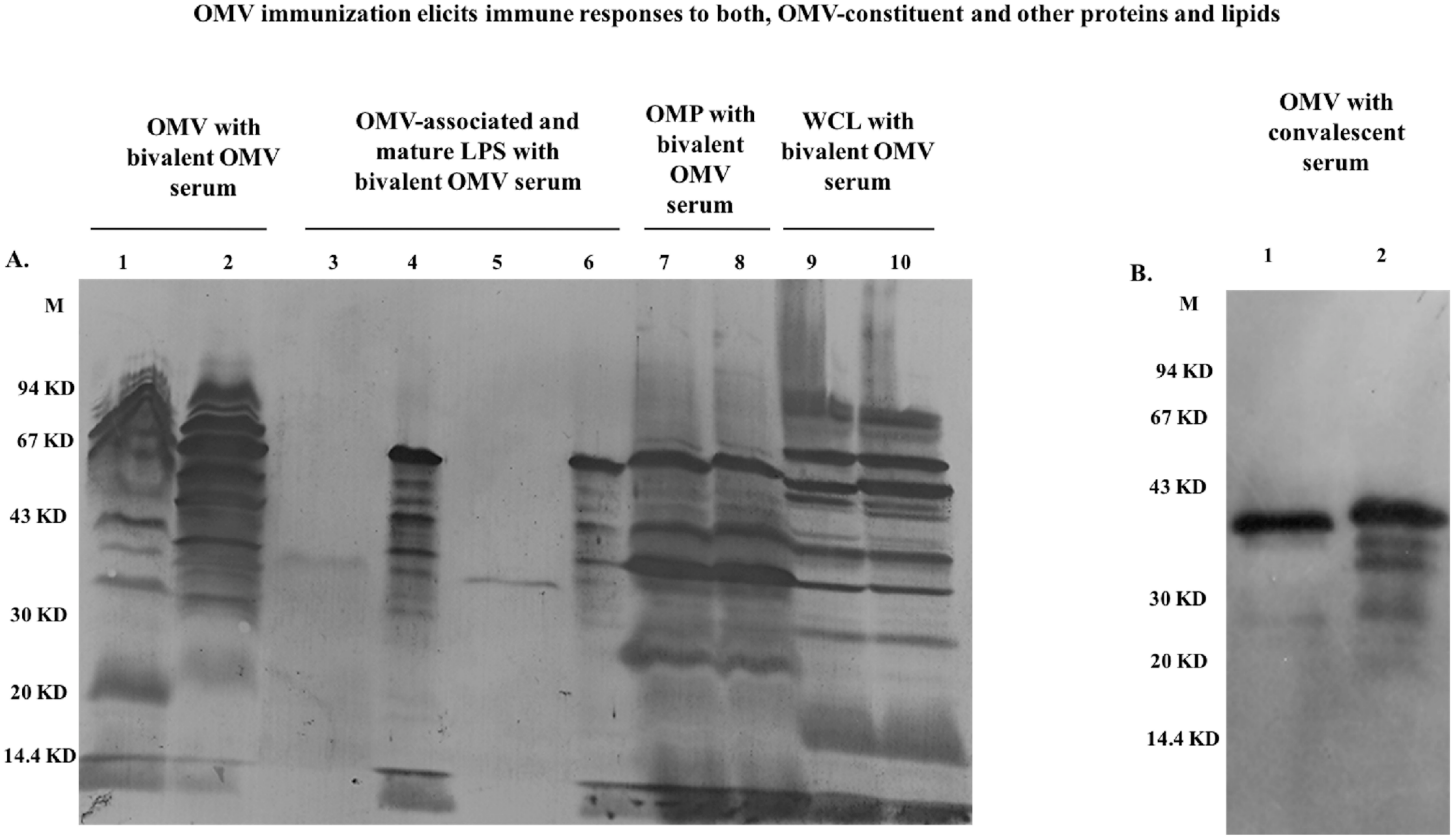
Representative immunoblot analysis against OMVs, proteinase K-treated OMVs, isolated OMPs, whole cell lysate (WCL) using the same anti-OMV mouse serum and immunoblot of OMVs using convalescent sera of two typhoidal strains. **A.** Immunoblot against each component of the OMVs, LPS, OMPs and WCL of the bivalent formulation probed with 28^th^ day’s anti-bivalent OMVs serum from a single mouse. Lane M: Pre-stained molecular weight marker (Bangalore Genei), Lane 1: *S*. Typhi, Lane 2: *S*. Paratyphi A OMV. Lane: 3, 5: OMV-associated LPS of *S*.Typhi and *S*. Paratyphi A, respectively. Lane 4, 6: Isolated native LPS from *S*.Typhi and *S*. Paratyphi A, respectively. Lane: 7, 8: *S*.Typhi and *S*.Paratyphi A OMPs. Lane: 9, 10: *S*. Typhi and *S*.Paratyphi A WCL. **B.** Immunoblot analysis against each component of the OMVs of the bivalent formulation using convalescent sera of mice. Lane: 1 and 2: *S*.Typhi and *S*.Paratyphi A OMVs.

In immunoblot analysis, immune-dominant bands were seen using anti-OMVs serum (Fig 3A). Lane 1 and 2 shows immune-dominant bands from OMVs separated by SDS-PAGE. A range of proteins, starting from 20 and ending near 90 KD regions, were found to be immunogenic. Immune-dominant bands against OMV-associated and native, mature LPS was seen in lane 3, 4, 5 and 6. Bivalent OMV-immunized mice serum was also used to detect immunogenicity of mature LPS from these two strains by dot blot analysis (S12 File). Although the OMV-associated LPS and native LPS are structurally different, but they both were recognized by the polyclonal anti-OMV serum raised in mice. In case of purified outer membrane proteins of *S*.Typhi and Paratyphi A against OMV-immunized sera, several outer membrane proteins of *S*.Typhi and Paratyphi A were found to be immunogenic in nature (lane 7, 8). The last two lanes (lanes 9 and 10), are showing the immunogenic nature of our immunogen against whole cell lysate isolated from these two strains. We found a number of proteins to be immunogenic in nature. To detect immunogenic proteins of OMVs, we also generated convalescent sera in mice by infecting them separately with wild type strains of *S*. Typhi and Paratyphi A intra-peritoneally. We did immunoblot using these immunized sera against two OMVs and we found immune-dominant bands in the region of nearly 20–40 KD (Fig 3B). From this study, we can conclude that both OMVs contained different immunogenic proteins and also different immunogenic outer membrane proteins.

### Induction of cell-mediated immune response—Cytokines from BMDCs, splenic cells and BMDC-splenic CD4 T cell co-culture and FACS analysis

#### i. Production of Th1-Th17 immune response provoking cytokines were evident ex vivo

**Fig 4:**
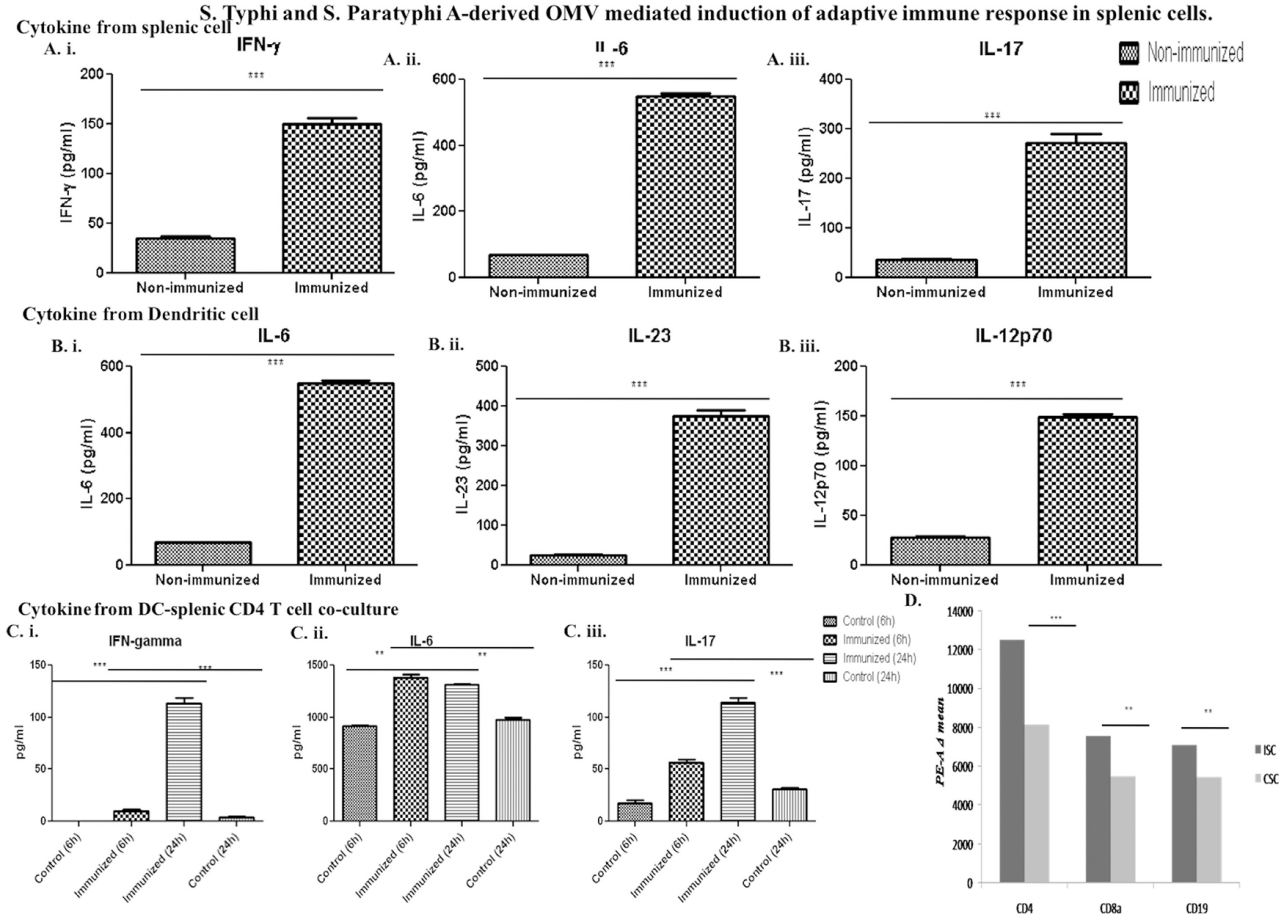
Bivalent OMVs induces the production of Th1 and Th17 polarizing cytokines. A. Splenic cells after immunization and B. In naïve matured *ex vivo* stimulated BMDCs. Splenic cells collected from immunized mice and BMDCs were collected from non-immunized and kept in GM-CSF supplemented media until their maturation and then were treated with 10 μg/ml bivalent OMV antigens. After 24 hours, levels of IFN-γ, IL-6 and IL-17 from splenic cells and IL-6, IL-23, IL-12p70 were measured by ELISA (n = 3, each group). **C.**BMDCs from naïve mice were collected and incubated until maturation and splenic CD4 T cells from immunized and naïve mice collected and co-culture with the matured BMDCs. Stimulation of these co-cultured cells with the same OMVs produced significant amounts of IFN-γ, IL-6 and IL-17 which were measured by ELISA (n = 3, each group)(*** P < 0.005). **D.** Up-regulation of CD4, CD8a and CD19 was shown in terms of PE-A values. (n = 3, each group) (*** P < 0.005).

After three successive oral immunizations, on the 35^th^ day, splenic cells were isolated from both immunized and non-immunized mice and were treated with bivalent OMVs for 24 hours *in vitro* at 37°C, in 5% CO_2_. Protein-level up-regulation of IFN- γ, IL-6 and IL-17 were found (Fig 4Ai, 4Aii and 4Aiii).

Matured dendritic cells were also found to be secreting significant amounts of IL-6, IL-23 and IL-12p70 (Fig 4Bi, 4Bii and 4Biii). Co-cultured matured BMDCs and splenic CD4 T cells were found to be secreting significant amounts of IFN-γ, IL-6 and IL-17 (Fig 4Ci, 4Cii and 4Ciii). All these results indicate a strong Th1-Th17-biased immune response in immunized mice following OMV immunization.

#### ii. FACS–up-regulation of B- and T-cell specific surface markers indicates the activation of an adaptive immune response following OMV immunization in mice

We tested the changes in expression of CD4, CD8a and CD19 on splenic cells. The expression of CD4, CD8a and CD19 was greatly increased (S13 File, Fig 4D; Table 2). This result indicates up-regulation of both humoral and cellular arms of the immune response.

**Table 2:** Up-regulation of different surface markers by FACS.

	Markers	CSC			Mean	Δ mean from 3 animals	STDEV of mean	ISC			Mean	Δ mean from 3 animals	STDEV of mean		Difference in Δ mean	% up-regulation
1^st^ experiment	CD4	8022	8144	8654	8273.33	8151.77	105.44	12908	12304	12475	12562.33	12535.11		62.14	4383.33	34.96
	CD8a	5754	5123	5671	5516	5503.44	18.93	7668	7126	7892	7562	7551.33		32.02	2047.88	27.11
	CD19	5475	5124	5612	5403.66	5456.55	45.84	7099	6830	7109	7012.66	7089.55		83.27	1633	23.03
1^st^ repeat	CD4	7900	8044	8311	8085			13123	12401	12213	12579					
	CD8a	5500	5256	5689	5481.60			7789	7098	7843	7576.66					
	CD19	5322	5221	5900	5481			7121	6900	7213	7078					
2^nd^ repeat	CD4	8245	7810	8236	8097			12823	12132	12437	12464					
	CD8a	5409	5651	5478	5512.60			7956	7245	7345	7515.33					
	CD19	5345	5145	5965	5485			7231	6846	7457	7178					

Values are means ± SD of triplicate biological and technical repeats.

### Protective efficacy study in intra-peritoneal challenge adult mice model

We have standardized the intra-peritoneal mice model of typhoidal *Salmonella*. We saw a clear dose-dependent response in terms of survival of the infected mice, with the LD_50_ value to be 2 x 10^7^ CFU/ml for both the challenge strains of *S*. Typhi and *S*.Paratyphi A (S14 File). Along with that, we have found strong association of dose-dependent response with colonization rate as well. In our time-dependent approach, we have found both typhoidal salmonellae strains to be present in the spleen and liver after 2–3 days.

After developing the mice model, we wanted to check how the immunogen was working. Before assessing the effectiveness of bivalent immunogen, we wanted to check the nature of protection in monovalent OMV-immunized mice. We found that, monovalent OMV-immunized mice were protected against homologous strains but not against heterologous strains (S15 File). Then we moved onto deciphering the effect of bivalent OMV.

**Fig 5:**
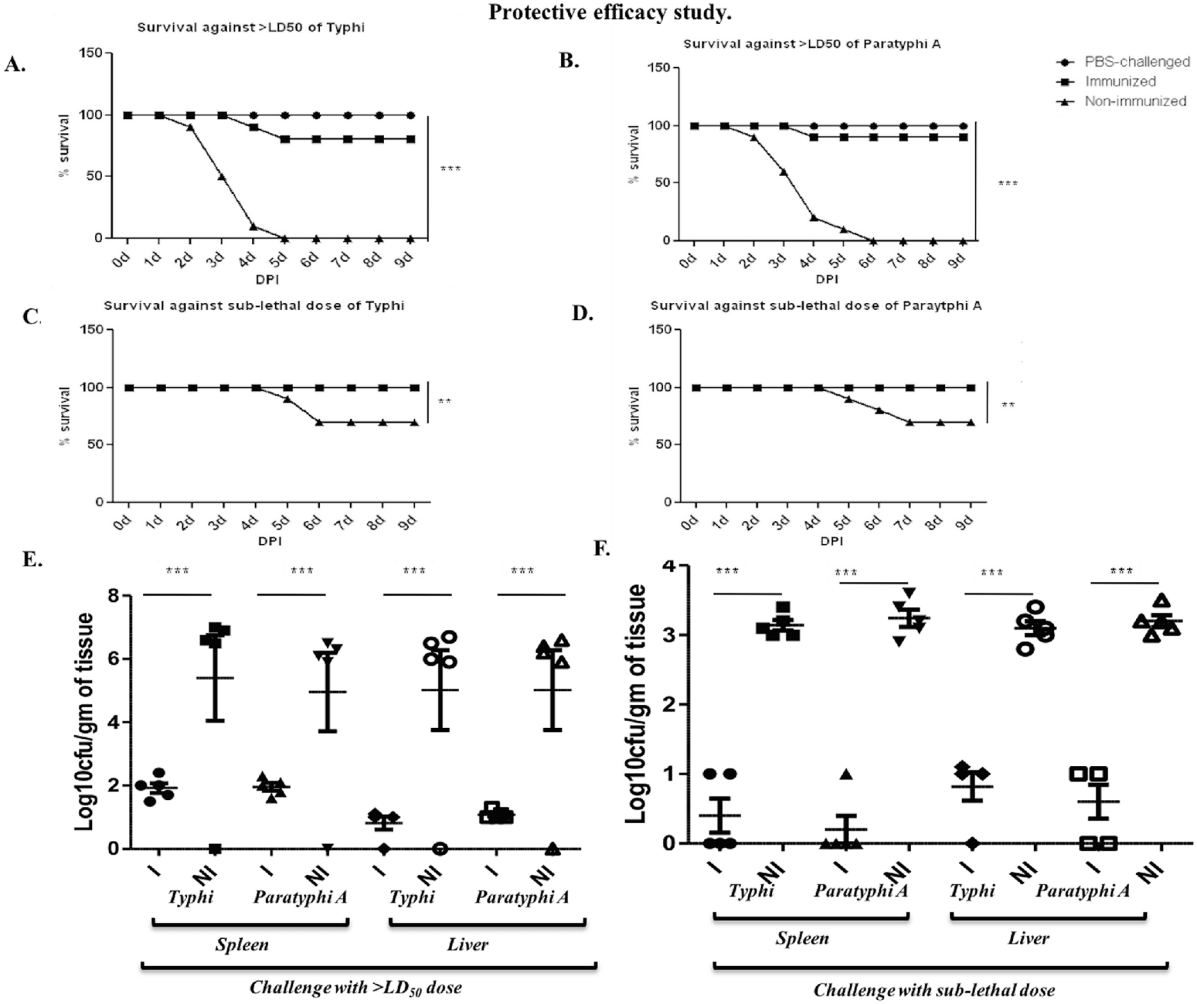
Immunization with the bivalent OMVs provides protection in adult mice model. Mice were immunized with 1:1 mixture of typhoidal OMVs using 25 μg total OMVs per dose and a three-dose immunization. Mice were then challenged with 1 x 10^8^ CFU/ml and 3 x 10^5^ CFU/ml of each challenge strains and observed for the period of survival for 9 days. **A., B.** Percent survival against *S*. Typhi and *S*. Paratyphi A lethal challenge and **C., D.** Percent survival against *S*. Typhi and *S*. Paratyphi A sub-lethal challenge. **E.** An infection with lethal dose of typhoidal strains caused a huge colonization of systemic organs after both *S*. Typhi and *S*. Paratyphi A infection. **F.** A lower level of colonization of systemic organs was seen in mice challenged with sub-lethal dose of infection.

In protective efficacy study, we used the same adult mice model. Immunized and non-immunized mice were intra-peritonealchallenged with the doses described in the methods. We kept a negative control which we termed PBS-challenged to eliminate any bias. In case of non-immunized mice, all the mice died within 3–5 days (Fig 5A and 5B). But, 80% and 90% immunized mice were survived after 9 days. The sub-lethal dose, on the other hand, was only able to kill 30% mice in non-immunized challenged groups (Fig 5C and 5D). All the immunized mice survived this infection. Negligible effect was observed in terms of change in body weight of the immunized mice. Colonization data suggests the immunogen’s potent nature against systemic spread and infection of typhoidal salmonellae in mice and it indeed was protecting the mice infected with both the doses of infection (Fig 5E and 5F).

Reduction in body weight was observed in non-immunized mice after both lethal and sub-lethal challenges (S16 File). Although much less reduction in body weight was observed after sub-lethal challenge, a higher thanLD_50_ dose caused significant change in body weight. Bivalent OMV-immunized mice were shielded from the infection that occurred in the non-immunized mice (Fig 5 and S16 File). At 3 DPI, we observed 105 –10^8^CFU/gm of infection in spleen, liver in case of non-immunized mice, whereas, 10^1^−10^2^ CFU/gm of infection in spleen, liver was the highest colonization found in immunized mice after higher than LD_50_ challenge (Fig 5E). After sub-lethal challenge lesser level of colonization was found as expected (Fig 5F). Colonization in small intestine (S17 File).

### Adoptive transfer of splenocytes and sera from immunized mice protects naïve mice from typhoidal salmonellae-induced lethal infection

To find out whether humoral or T-cell induced cellular immunity is important for the protection against *Salmonella*, we transferred serum and splenocytes via tail vein in non-immunized mice (S18 and S19 Files). We found both immunized serumand splenocytes had protected the naïve mice almost equally. We did survival assay of these mice against challenge bacteria and found it to be 70–100% protective in both cases after challenging them on day 0. We have also measured the systemic spread of the challenge strains in adoptive transferred naïve mice. Colonization in spleen and liver were found to be in the range of 1 x 10^6^–1 x 10^7^ CFU/gm of the respective organs in case of control or non-immunized mice (S18 File). Whereas, immunized sera and splenocytes recipient mice showed significantly lower colonization. We challenged the other adoptively transferred mice on day 7 and a slight increase in the colonization was seen (S19 File). This result suggested that protection against *Salmonella* depends on both humoral and cell mediated immunity.

### Mode of protection

#### Bivalent OMV-immunized mice sera agglutinate wild-type bacteria in vitro

**Fig 6:**
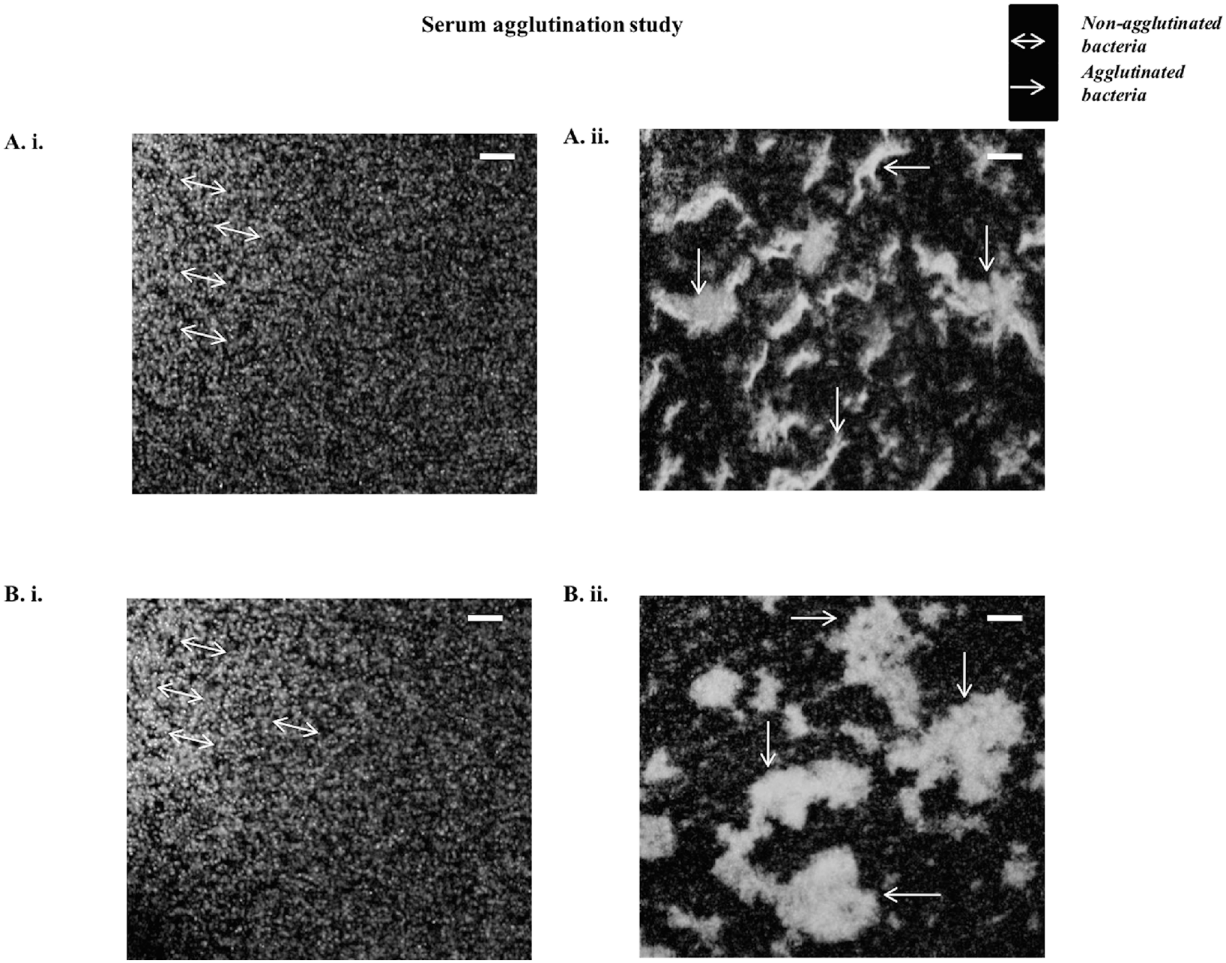
Heat-inactivated serum from bivalent immunized mice forms agglutination when comes in contact with the GFP-tagged clinical isolates of S. Typhi and Paratyphi A. **A.** i.*S*. Typhi treated with heat-inactivated non-immunized serum, **A.** ii. *S*.Typhi treated with heat-inactivated immunized serum. **B.** i. *S*. Paratyphi A treated with heat-inactivated non-immunized serum, **B.** ii. *S*. Paratyphi A treated with heat-inactivated immunized serum. Bar represents 10 μm.

To check if the LPS and Vi-polysaccharide specific antibody generated by OMVs immunization can detect the bacterial outer surface of both *S*. Typhi and Paratyphi A we did agglutination test in presence of OMVs immunized sera and non-immunized sera (heat-inactivated). We found non-immnunized sera treated wild-type bacteria did not show any agglutination (Fig 6Ai and 6Bi). On the other hand, visible clump or bacterial agglutination in immunized sera treated group under fluorescence microscope (Fig 6Aii and 6Bii). Specific arrows were being used to indicate both non-agglutinated and agglutinated bacteria. This result indicates that; our bivalent OMV-immunized sera can activate complement mediated killing or innate immune response.

#### Inhibition of S. Typhi and Paratyphi A motility and mucin penetration ability in vitro by antibodies from OMV- immunized mice

**Fig 7:**
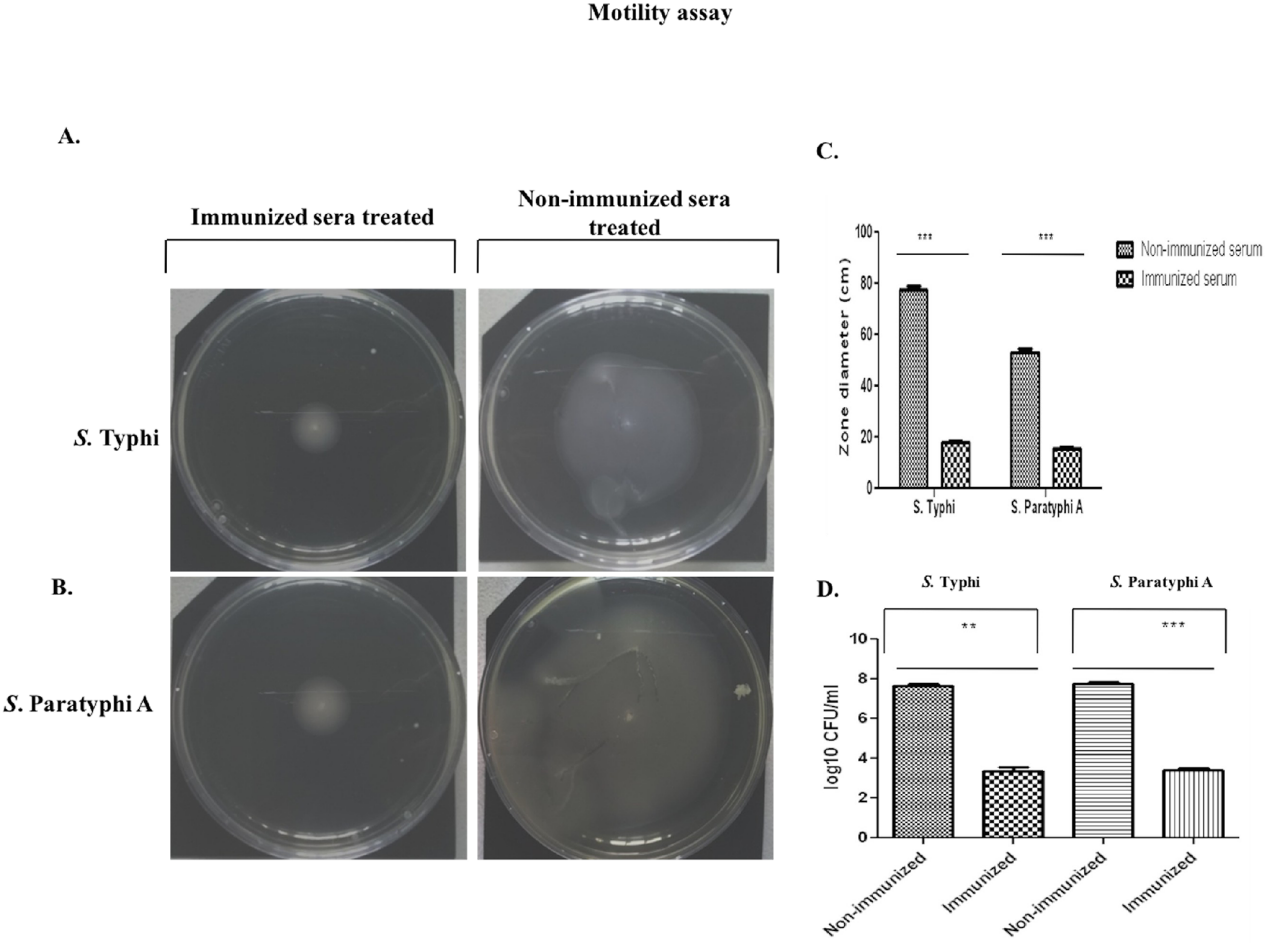
Serum from bivalent immunized mice inhibits S. Typhi and Paratyphi A motility. Serum from non-immunized or immunized mice was mixed 1:400 with PBS and was spreaded over the soft agar (0.3% agar) plates. Plates were dried and the plates were punctured at the center either with one colony of *S*. Typhi or with one colony of *S*. Paratyphi A and kept at 37°C for 24 hours. **A.** The spread of *S*. Typhi and **B.** *S*. Paratyphi A was **C.** Significantly reduced in case of serum collected from immunized mice **D.** Bacteria treated with non-immunized or immunized serum were loaded on top of the 1 ml mucin column and allowed to penetrate. Immunized serum agglutinated bacteria showed reduced ability to penetrate mucin.

In our next experiment, we did heat–inactivation of immunized sera and non-immunized sera to deactivate the complement and incubated heat-inactivated immunized and non-immunized serum with *in vitro* grown *Salmonella* strains. We found significant inhibition of the *Salmonella* motility. Immunized serum, compared to non-immunized serum showed significant difference in the zone of spread of bacteria (Fig 7A, 7B and 7C). This result suggests that our bivalent formulation is indeed resulted in agglutination of bacteria which could be a way towards protective nature of this immunogen.

Next, we investigated mucin penetration ability of both typhoidal *Salmonella* strains in presence of heat-inactivated immunized and non-immunized sera. We found immunized serum-treated bacteria showed lesser mucin penetration ability in the range of 2 x 10^3^ CFU/ml to 3 x 10^3^ CFU/ml. Whereas, the ability of penetration by non-agglutinated bacteria were much higher, in the range of 3 x 10^7^ CFU/ml to 4 x 10^7^ CFU/ml (Fig 7D). This data indicate that OMVs specific sera significantly inhibit motility which can inhibit mucin penetration that can help to inhibit colonization or invasion into the epithelial cells.

### OMVs specific serum induces opsonization in peritoneal macrophage

The bacteria-killing ability of the isolated immunized sera was evaluated in peritoneal macrophages. Bacteria incubated with mouse anti-bivalent OMV sera were killed more efficiently by the macrophages than that of the bacteria incubated with non-immunized sera. Bivalent sera had efficiently increased phagocytosis ability, thus increasing the bacteria killing property (Table 3).

**Table 3:** Opsonization activity of immunized and non-immunized sera.

Bacteria	Treated with	Mean CFU x 10^5^ / well		Source or reference
		Incubation condition		
		0 minute	60 minutes	
*S*. Typhi C-6.946	Immunized serum	2.3±0.3	9.3±0.7**	This study
	Non-immunized serum	2.1±0.5	50.5±0.1	
*S*.Paratyphi A BCR 148	Immunized serum	2.5±0.2	10.4±0.8**	This study
	Non-immunized serum	2.6±0.4	53.6±0.7	

Values are means ± SD of triplicate samples.

**p < 0.005 (highly significant) when compared with the respective result of non-immune sera.

All the above results suggest that both humoral and cellular immunity confer the protective effect of *S*. Typhi and *S*. Paratyphi A bivalent OMVs-immunization.

### Discussion

There is an urgent need for a suitable broad spectrum typhoidal *Salmonella* vaccine [1]. Still no such vaccines have the ability to provide a complete protection against *S*. Typhi and Paratyphi A together. Moreover, no licensed vaccine is available for paratyphoid fever. One of the major bottlenecks for the development of such vaccine is lack in the identification of protective antigens. Recent study showed that secreted or surface-associated antigens of *Salmonella* can induce strong protective humoral and cell mediated immune response [42]. Surface associated antigens are more protective than internal antigen because of physical association with PAMPs such as lipopolysaccharide that can activate both innate and adaptive immunity.

Recent studies demonstrated that OMVs of gram-negative bacteria are one of the promising candidate vaccines [43, 44, 45, 46, 47, 48]. The composition of OMVs reflects outer membrane structure of the bacteria. So, we have isolated the OMVs from *S*. Typhi and Paratyphi A and characterized them. We found that both the OMVs contained LPS, different outer membrane, inner membrane, cytosolic and virulence proteins. Our study indicates that, OMV-constituents bridge their potent immunogenic nature with protective immuneresponse against enteric fever. To develop a complete broad range of protective immune response against both *S*. Typhi and Paratyphi A, we have designed a bivalent OMV-based vaccine and measured protective immune response in mice model. After oral immunization of three doses of bivalent *Salmonella* OMVs immunogen, we found antigen specific antibody responses, namely, anti-LPS, anti-Vi polysaccharide, anti-OMPs, anti-WCL. This result indicated that our formulation was immunogenic in nature. Furthermore, our data also indicates that OMVs are more immunogenic than heat-killed immunogen generating higher response for the production of antigen specific antibodies. Our results have also suggested that OMVs have an ability to induce humoral as well as cell mediated immune response. We found that after oral immunization, the populations of CD4 T and CD8 T cells in spleen were significantly increased. That data indicates that our immunogen activates T cell-mediated immune response. From this study, we can conclude that our immunogen significantly induce adaptive immune response; both humoral and cell mediated immune responses which are very crucial for generating long term protective immune response. The notion that, generation of both humoral and cellular arms of the immune system is necessary for the generation of long-term immune response was supported by previous studies as well [49, 50, 51]. LPS and Vi-polysaccharide are crucial antigenic components of typhoidal vaccine [52, 53]. Both LPS and Vi-polysaccharides are T–independent antigens [54]. Vi-polysaccharide typhoidal vaccine generates Vi-polysaccharide specific IgG by T-independent manner. For this reason, Vi-polysaccharide based vaccine was unable to generate significant long term protection (only 55% of the population was protected) [55]. Further studies showed that conjugated Vi-polysaccharide vaccine is more effective to generate long term protection than the native one [56, 57]. Vi-polysaccharide was conjugated with tetanus toxoid which induces significant T-cell depended immune response [56, 57]. Recent study showed that different outer membrane proteins, such as, OmpF, OmpC, OmpS1 and OmpS2 of *S*.Typhi are immunogenic and acts like adjuvant, they also induce T-dependent immune response [58]. Our proteomics data and immunoblot result showed that OMVs contained several outer membrane proteins and they are immunogenic. This data indicates that proteins of OMVs act as an adjuvant which can significantly induce long term Vi-polysaccharide and LPS specific antibodies in a T-depended manner.

On the other perspective, cell-mediated immune responses are necessary to prevent intracellular *Salmonella* infection and spreading [59]. In our study, we found our bivalent immunogen to be significantly producing Th1 and Th17 polarizing cytokine which indicate activation of innate and cell mediated immune response [60, 61, 62]. Our result showed that our immunogen significantly induces Th1 and Th17 mediated immune response; generating significant amount of IFN-γand IL-17. BMDCs-splenic CD4 T cell co-culture could actually mimic the condition of antigen presentation and up-regulation of different arms of the immune system. Elevation of IFN-γ and IL-12 indicates that the immune response may activate both CTL and Th1-response. IL-6 has a reputation of bridging the gaps between innate and adaptive immune response as well [63]. Up-regulation of CD4, CD8a and CD19^+^ cells indicate the activation of both B- and T-cells of the immune system [64]. Th1 cell mediated immune response is very crucial for *Salmonella* specific protective immune response [65]. A study showed that less IFN-γ producing mice are more susceptible to *Salmonella* Typhimurium infections compared with wild-type mice [66]. IFN-γ has a distinct role to activate macrophage for the clearance of invasive bacteria [67, 68]. Furthermore, IL-17-producing Th17 cells are necessary for vaccine-induced protection against bacterial infection by enhancing neutrophil recruitment to infection sites and direct bacterial killing property [69]. Both IL-17 and IL-22 are produced in the intestinal mucosa early after oral *Salmonella* infection [70]. After *Salmonella* infection, IL-17 deficient mice demonstrate a modest increase in bacterial dissemination, suggesting that IL-17 contributes to the maintenance of the mucosal barrier [71]. Indeed, depletion of intestinal CD4^+^ T cells that accompanies simian immunodeficiency virus infection selectively blunted the intestinal IL-17 response in rhesus macaques, allowing increased translocation of *Salmonella* to the mesenteric lymph nodes and spleen [72]. Thus, although Th1 cells are critical for bacterial clearance in systemic tissues, Th17 cells most likely play an important additional protective role in preventing bacterial dissemination from the intestine.

In the protective study, we have challenged the immunized and non-immunized mice intraperitoneally with lethal dose of lethal wild type *Salmonella* Typhi and Paratyphi A strains. This mice model is wildly used and most acceptable mice model from others like iron overload model due to the reproducibility and easy for the vaccine study for Typhi infection (32). We found almost 80–90% survival rate in immunized group and the bacterial burden in different organs of immunized group were significantly less in compare in immunized group in compare with non-immunized group after challenge. We can conclude that our formulated immunogen can provide broader range of protection against typhoidal fever.

Furthermore, we have checked the effect of both humoral and cell mediated immune response on protective efficacy by adoptive transfer of serum and spenocytes [73, 74]. This finding suggested that both humoral and cell mediated immune responses are crucial to induce protection against typhoidal *Salmonella* infection. Different serum transfer studies suggested that *Salmonella* specific antibodies are essential to complement fixation or opsonization of free bacteria [75]. Recent study showed that B cell-deficient mice did not produce Th1 and protective immune response after oral challenge with wild type *S*. Typhimurium strains and protective immune response cannot be restored by adoptive transfer of *Salmonella* specific antibodies [76]. Previous study also showed that B cells function as antigen presenting cells and an important source of inflammatory cytokines during *Salmonella* infection [77, 78]. So, both B and T cells are essential for *Salmonella*-specific immunity. Furthermore, much less colonization in the liver of immunized animals could be a result of neutrophils playing their part in providing protection [79]. In future, we will focus on which immune responses (B or T cells) is essential for clearance of diseases burden using different gene knock-out mice for direct evidence.

Here, we have further checked the mode of action of OMV specific antibody on *S*. Typhi and Paratyphi A. We found that OMV specific sera can agglutinate both *S*. Typhi and Paratyphi A which indicates the activation of complement mediated killing [80]. Agglutination was found to be occurring even after heat inactivation of complement in the sera which then helped in the inhibition of motility. Our result showed that OMV specific antibody significantly inhibits motility of *S*. Typhi and Paratyphi A. Bacterial motility is very crucial for penetrating the mucin layer, attachment and invading the intestinal epithelial cells [81]. Recent studies showed that *V*. *cholerae* OMV specific antibody significantly reduce adherence by inhibiting *V*. *cholerae* motility [82]. So, our data indicate that our immunogen also induced protective immune response by blocking initial bacterial entry via intestinal epithelial cells. Moreover, we found that typhoidal *Salmonella* OMV specific antibody enhance the macrophage mediated opsonization for killing the invasive strain. Overall our data suggest that our immunogen significantly induce first line defense of immune system to block and for clearance of typhoidal *Salmonella* infection.

## Conclusions

*S*. Typhi and *S*.Paratyphi A naturally secrets OMVs during their growth cycle. These OMVs are rich in outer-, inner-membrane associated and cytosolic proteins as well as LPS. 1:1 mixture of these typhoidal *Salmonella* OMVs is immunogenic in nature. Up-regulation of different cytokines and surface marker expression proves the elevation of both humoral and cellular arms of the immune response in mice model. These two arms of the immune response play together in providing protection in adult intra-peritoneal mice model. This bivalent formulation can be used in future in phase-I trial.

## Supporting information

S1 File Characterization of the strains used based on PCR and serology. All strains were characterized by both serotyping and PCR-based methods. Specific primers and anti O- and H-antigens were used for this purpose.
(TIF)

S2 File Characterization of the strains used based on their antibiotic susceptibility. Antibiotic susceptibility pattern was tested by Kirby-Bauer disc diffusion assay.
(TIF)

S3 File Schematic of immunization and challenge regimen. Mice were immunized by oral gavage on day 0 and then two subsequent booster doses follow as stated. Mice were then challenged on day 35 via intra-peritoneal infection model. Blood were drawn as shown by the dotted arrows.
(TIF)

S4 File MALDI-TOF/TOF spectrum of outer membrane vesicles (OMVs) isolated from *Salmonella* Typhi C-6953. (TIF)

S5 File MALDI-TOF/TOF spectrum of outer membrane vesicles (OMVs) isolated from *Salmonella* Paratyphi A C-6915. (TIF)

S6 File Hypothetical proteins present in *S*. Typhi OMVs. (TIF)

S7 File Hypothetical proteins present in *S*. Typhi OMVs. (TIF)

S8 File Hypothetical proteins present in *S*. Typhi OMVs. (TIF)

S9 File Hypothetical proteins present in *S*. Typhi OMVs. (TIF)

S10 File Hypothetical proteins present in *S*. Paratyphi A OMVs. (TIF)

S11 File Hypothetical proteins present in *S*. Paratyphi A OMVs. (TIF)

S12 File Dot blot and TCA precipitation analysis. A. Dot blot analysis against extracted LPS from two typhoidal strains. Lane 1: *S*. Typhi native LPS, Lane 2: *S*. Paratyphi A native LPS. Here, 1, 2, and 3 denotes three different concentrations of LPSs against which the dot blot analysis was performed. B.TCA precipitation of culture supernatant. Lane 1: *S*. Typhi culture supernatant, Lane 2: *S*. Paratyphi A culture supernatant.
(TIF)

S13 File FACS analysis was carried out after extracting the whole spleen. 50,000 events in total population were taken in each and every case and the mean value of area under PE in P2 population was taken into account whilst calculating the result. **i.** Unstained cells, **ii., iii., iv.** Up-regulation of CD4, CD8a and CD19, respectively in immunized and control mice’s spleen. **v.** REA-cloned Iso-Type Control (ITC) for all three markers.
(TIF)

S14 File Determination of LD50 value for challenge strains. 6 mice were per group were challenged with different concentrations of bacteria. Mice were observed for 7 days. The effective dose for LD50 or 50% mortality rate was found to be 2 x 10^7^ CFU/ml for both the challenge strains.
(TIF)

S15 File Comparative data of colonization between non-immunized and monovalent OMV-immunized adult mice, after challenging with wild type strains. **A**., **B**. Colonization in spleen and liver in *S*. Typhi OMV-immunized mice were less when they were challenged with *S*. Typhi. **C**. **D**.Colonization in spleen and liver in *S*. Paratyphi A OMV-immunized mice were less when they were challenged with *S*. Paratyphi A. In both cases,monovalent OMV immunization could not be able to hinder the infection from a heterologous strain.
(TIF)

S16 File Comparative data of change in body weight between non-immunized and immunized adult mice, after challenging with two wild type strains. **A**., **B**. A drastic change in body weight was seen in non-immunized mice challenged with >LD50 dose of infection. **C**. **D**. A lower level ofchange in body weight was seen in mice challenged with sub-lethal dose of infection.
(TIF)

S17 File Comparative data of bacterial load in small intestine. Three mice per group were challenged either sub-lethal dose or with greater than LD50 dose. Bacterial colonization in the small intestine was estimated since 3DPI until 5DPI. Higher colonization was observed in mice group challenged with greater than LD50 dose.
(TIF)

S18 File Comparative data of passive protective efficacies on 0^th^day challenge following adoptive transfer. Both B- and T-cell mediated immune response induced by OMVs is necessary for the protective immunity to bacterial infection. Splenocytes (1 x 10^6^) and serum (100 μl) were injected via the tail vain. One group of mice was challenged with 2 x 10^7^ CFU/ml of heterologous strains of bacteria after two hours of the adoptive transfer and kept for three days. **A**., **B**. Systemic infection of *S*. Typhi in spleen and liver was observed in the early group 3 days post infection. Similarly, **C**. **D**. Systemic infection of *S*. Paratyphi A in spleen and liver was observed 3 days post infection.
(TIF)

S19 File Comparative data of passive protective efficacies on 7^th^ day challenge following adoptive transfer. This group was challenged with the same dose as mentioned above after seven days of the adoptive transfer. **A**., **B**. Systemic infection of *S*. Typhi in spleen and liver wasobserved 3 days post infection. Similarly, **C**., **D**. Systemic infection of *S*. Paratyphi A in spleen and liver was observed 3 days post infection.
(TIF)
