# Supplementary figures and images for: Development of a novel S. Typhi and Paratyphi A outer membrane vesicles based bivalent vaccine against enteric fever
Source: PLoS One. 2018 Sep 14;13(9):e0203631. doi: 10.1371/journal.pone.0203631 (PMC6138408; doi:10.1371/journal.pone.0203631)

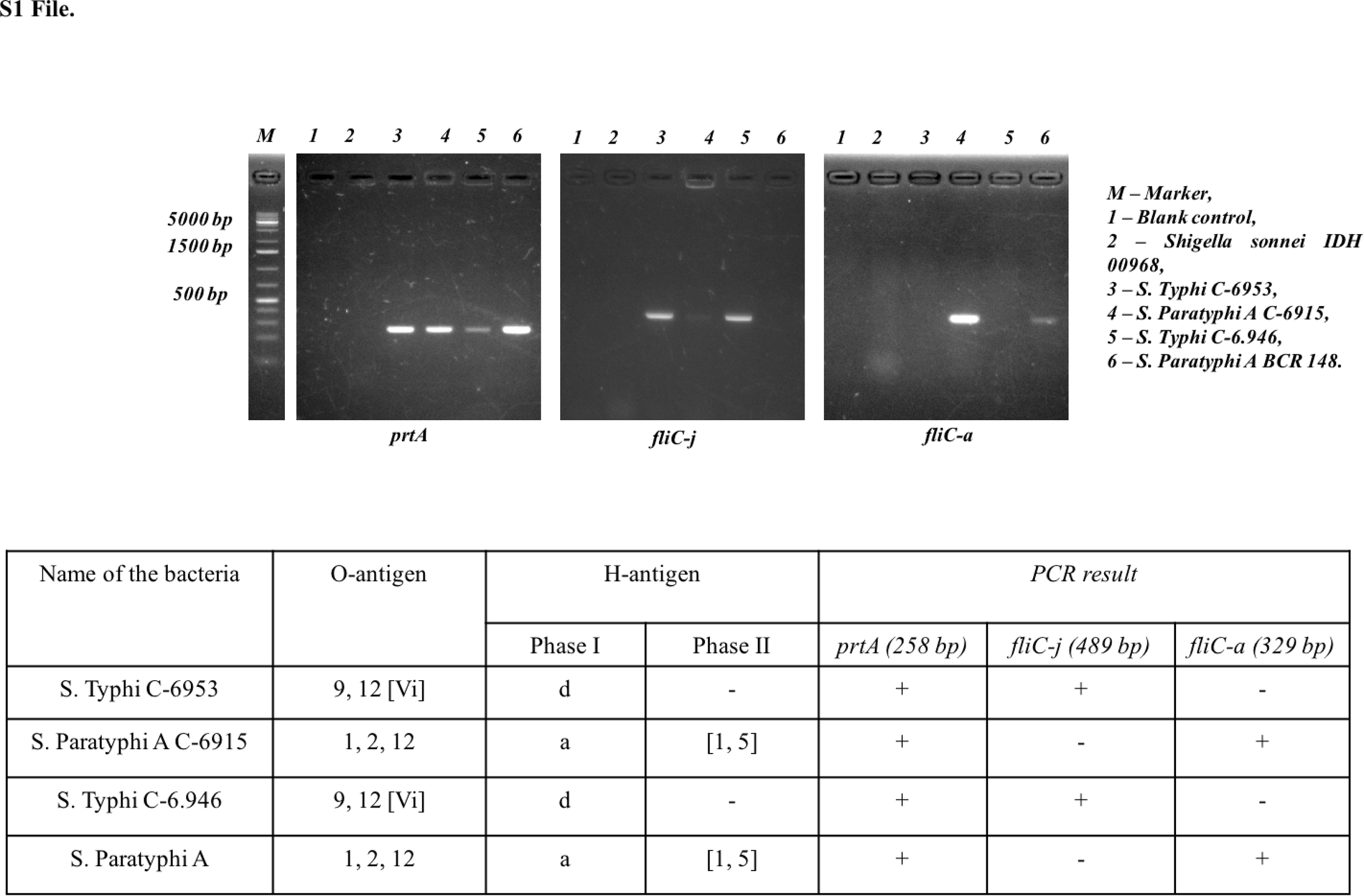

Supplement: S1 File — All strains were characterized by both serotyping and PCR-based methods. Specific primers and anti O- and H-antigens were used for this purpose. (TIF) [file pone.0203631.s001.tif]

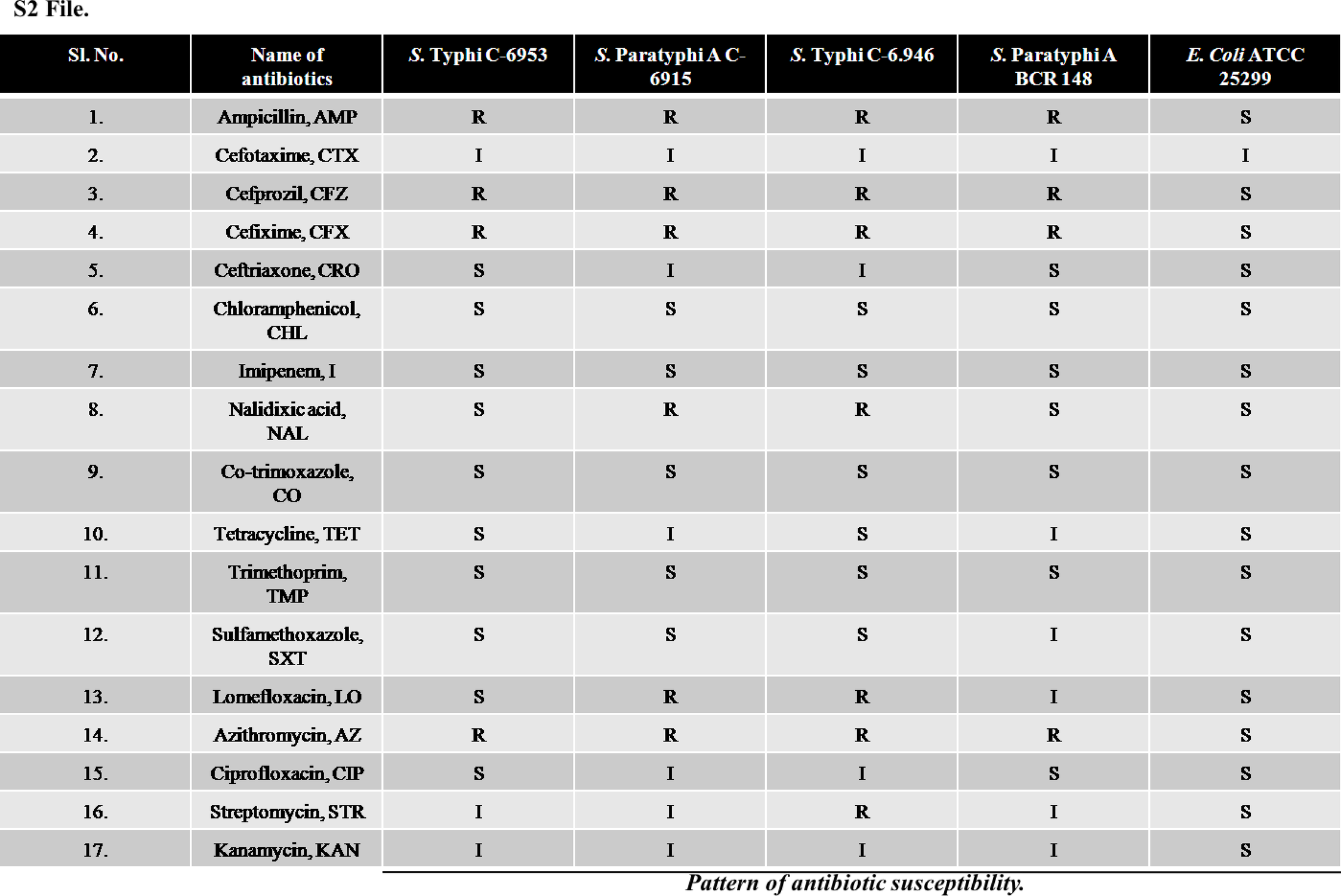

Supplement: S2 File — Antibiotic susceptibility pattern was tested by Kirby-Bauer disc diffusion assay. (TIF) [file pone.0203631.s002.tif]

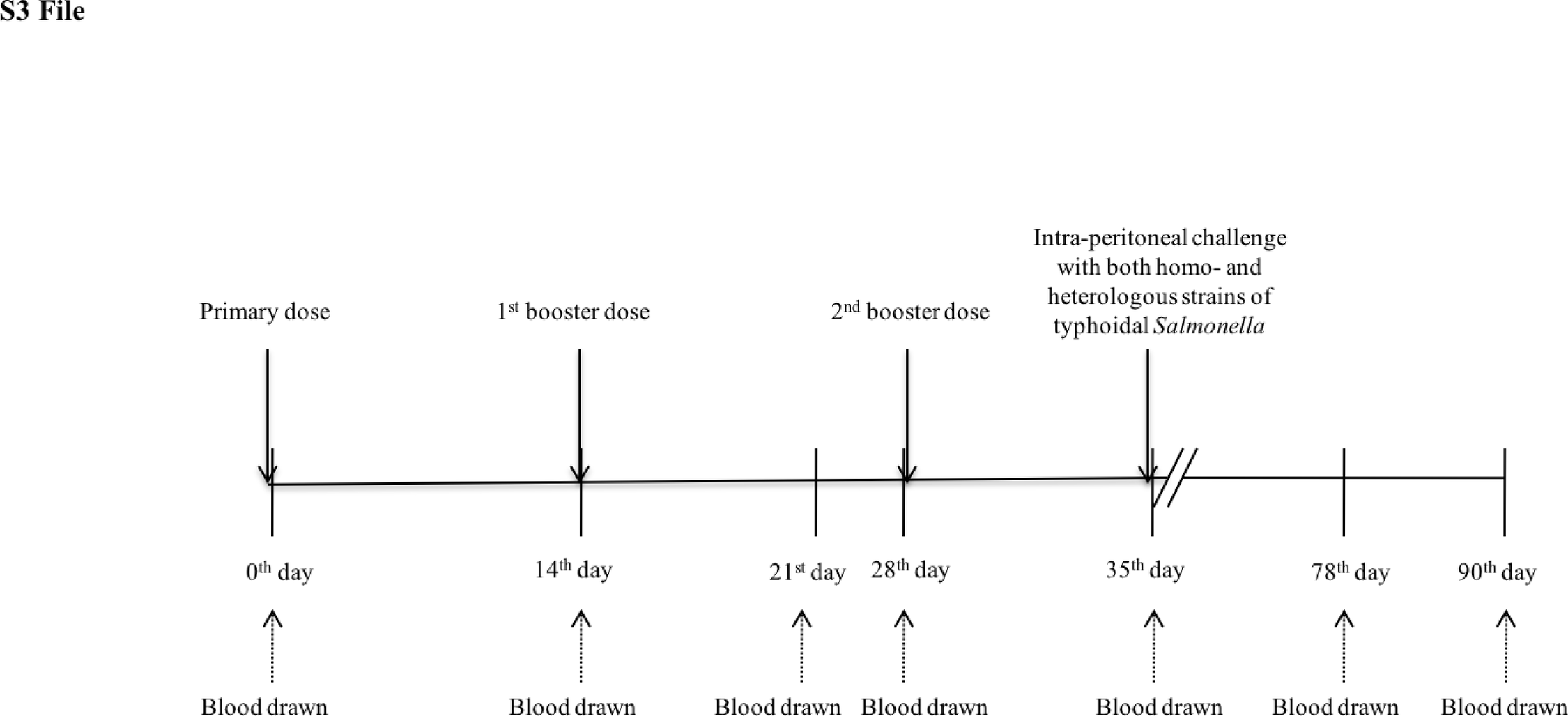

Supplement: S3 File — Mice were immunized by oral gavage on day 0 and then two subsequent booster doses follow as stated. Mice were then challenged on day 35 via intra-peritoneal infection model. Blood were drawn as shown by the dotted arrows. (TIF) [file pone.0203631.s003.tif]

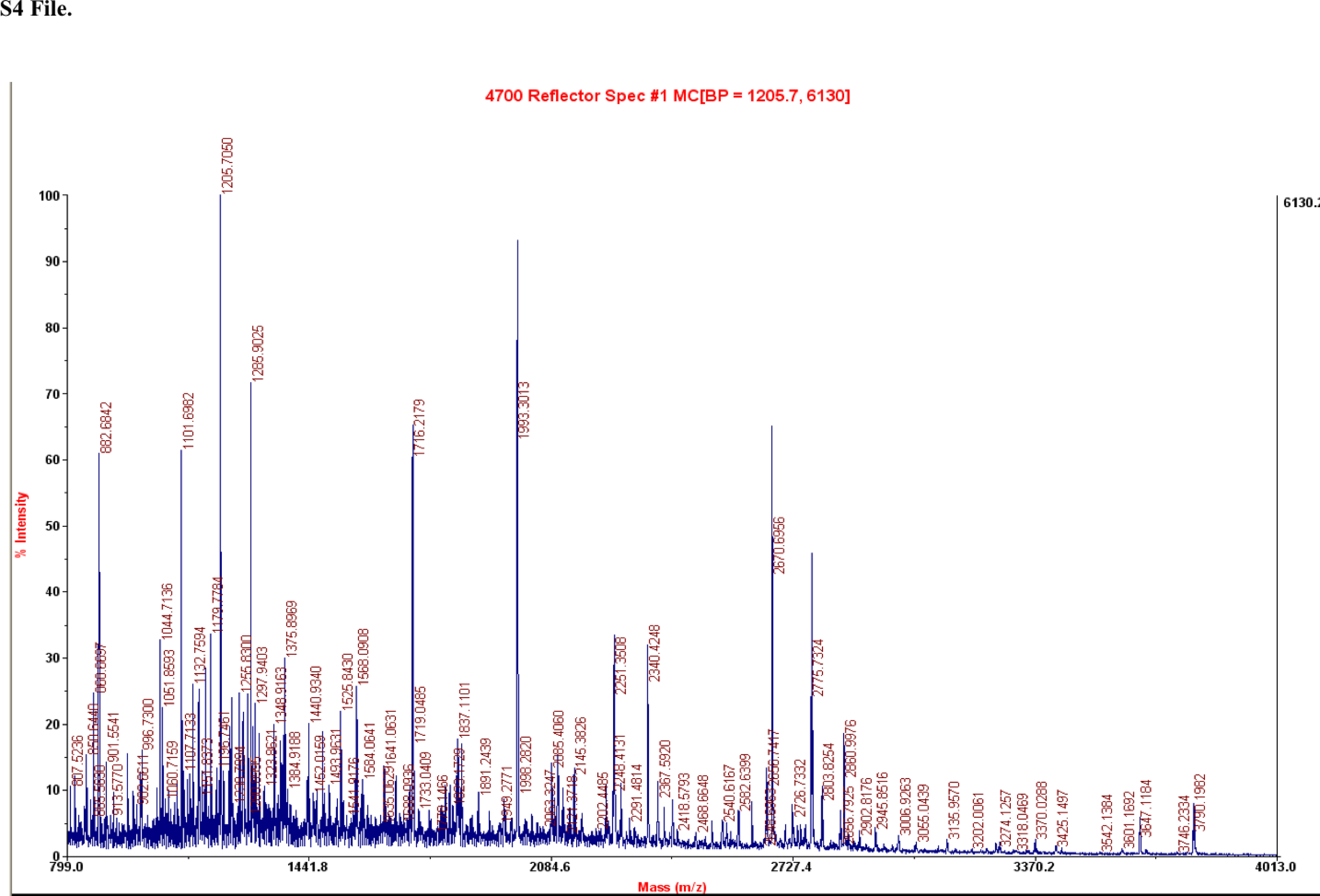

Supplement: S4 File — (TIF) [file pone.0203631.s004.tif]

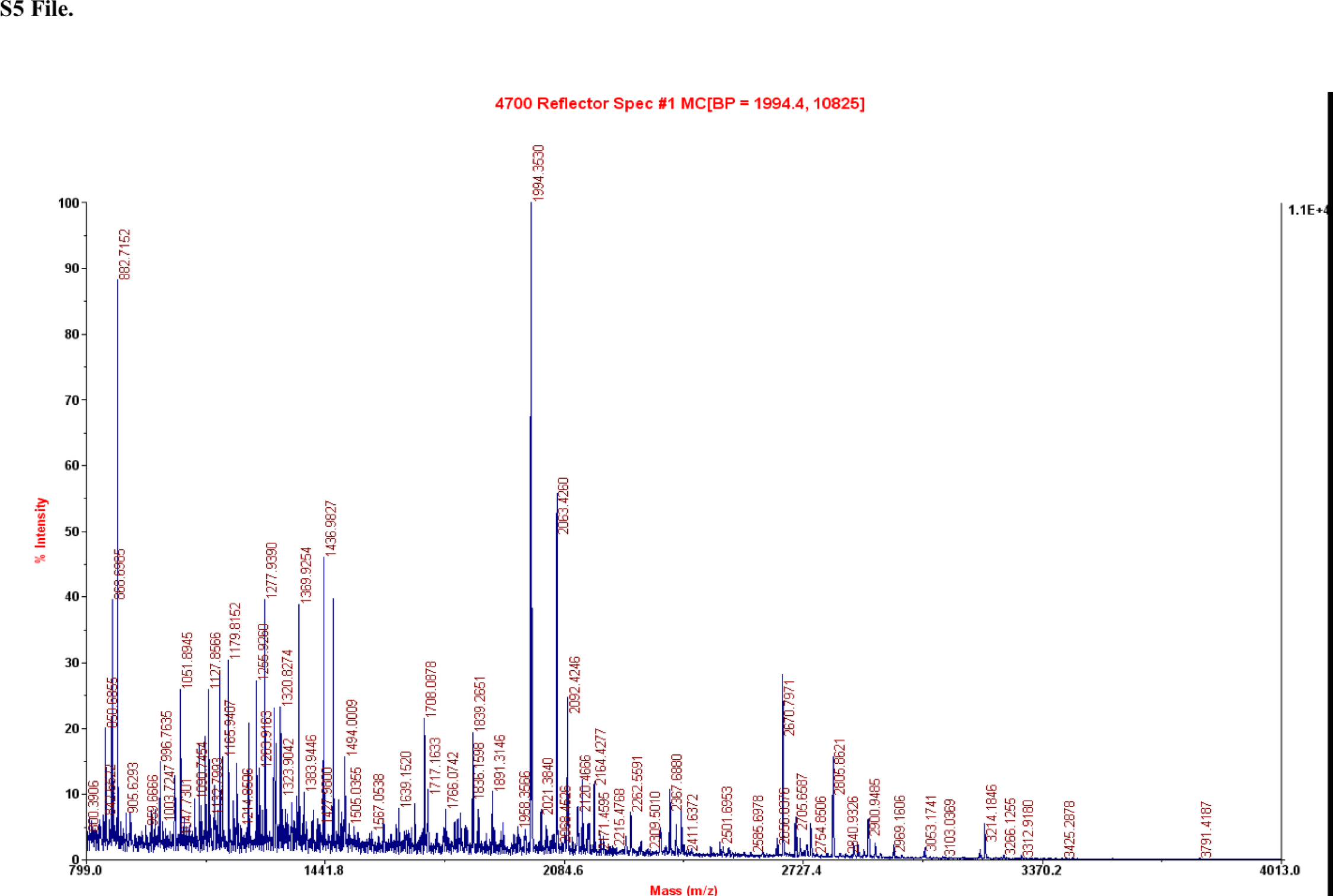

Supplement: S5 File — (TIF) [file pone.0203631.s005.tif]

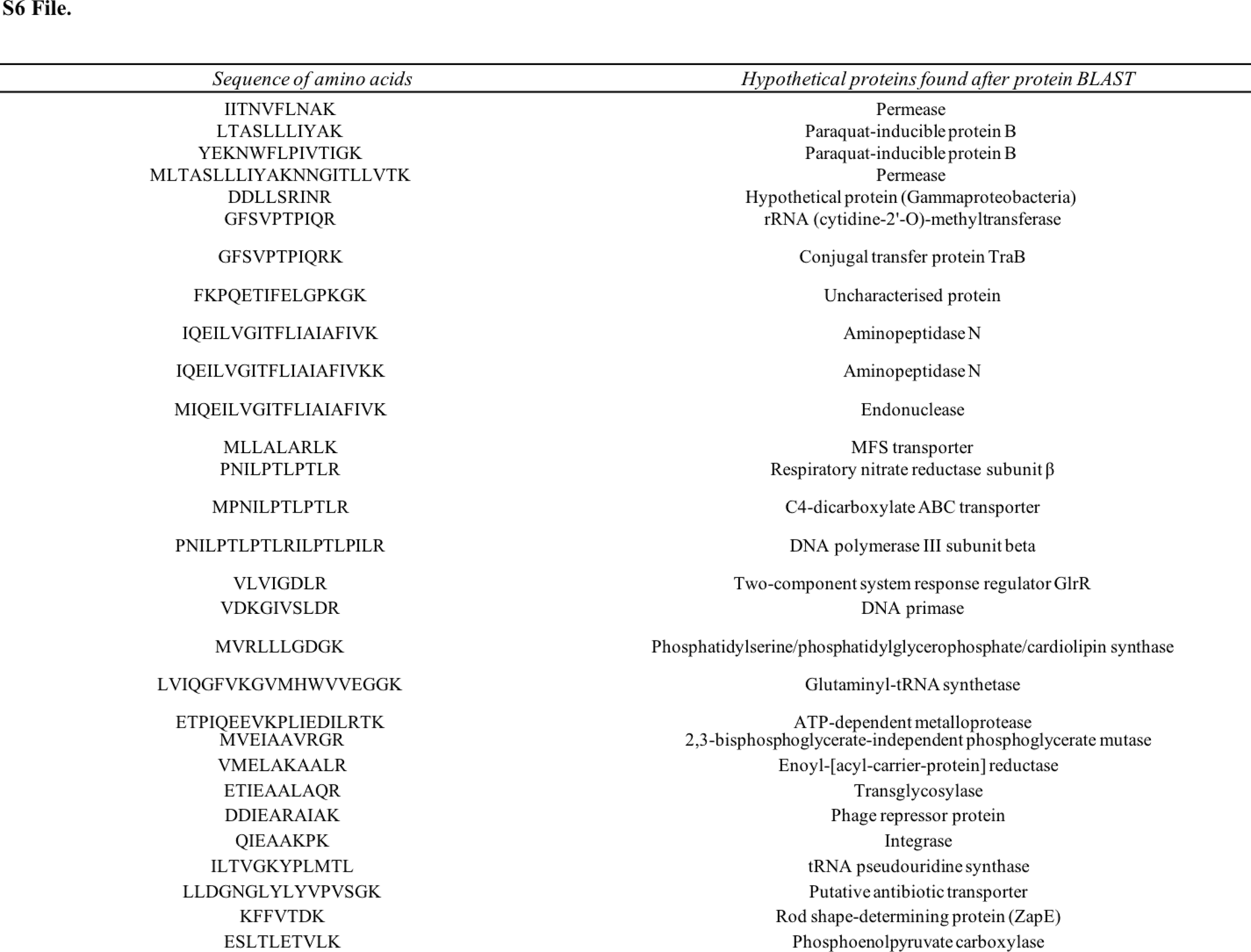

Supplement: S6 File — (TIF) [file pone.0203631.s006.tif]

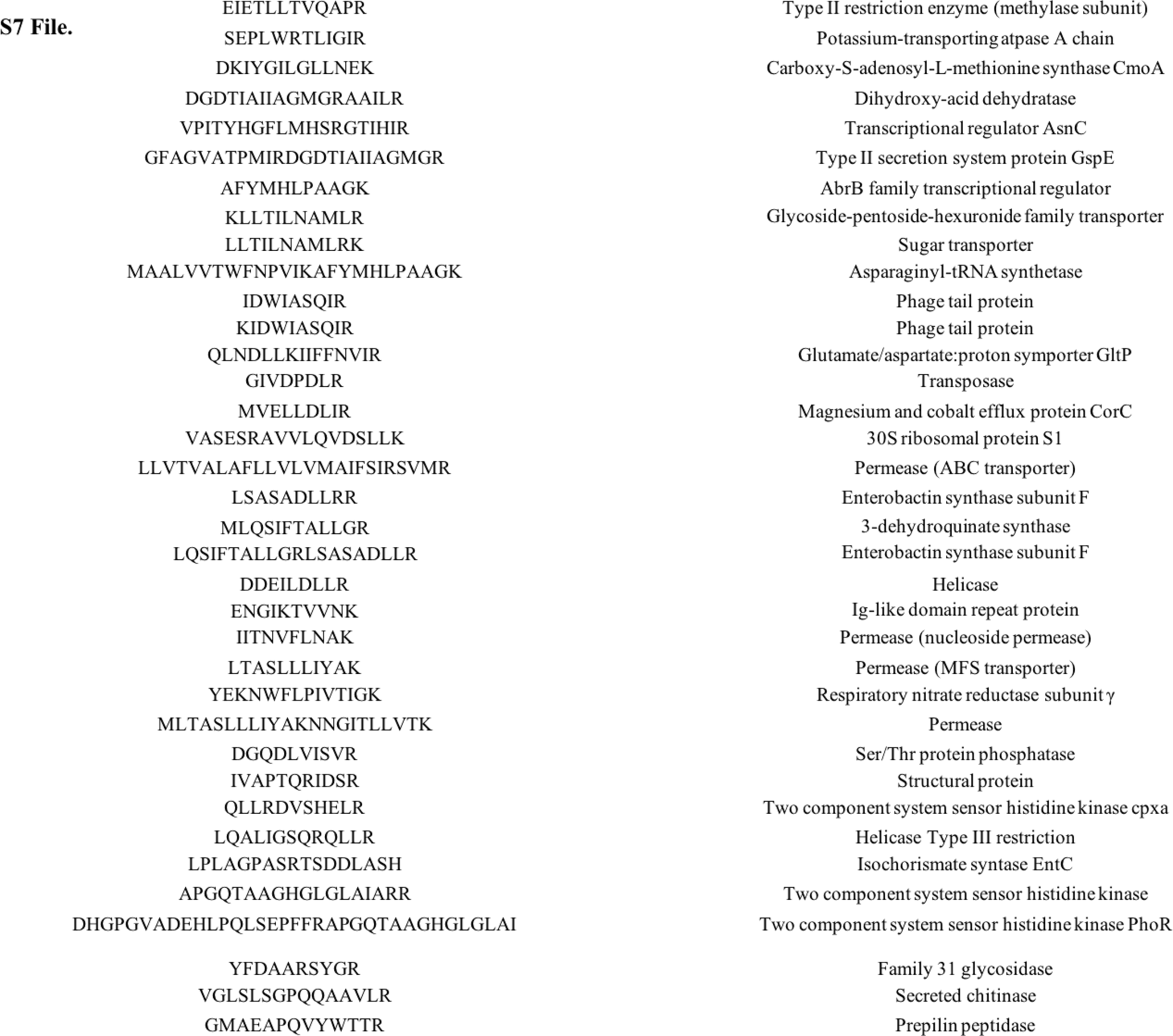

Supplement: S7 File — (TIF) [file pone.0203631.s007.tif]

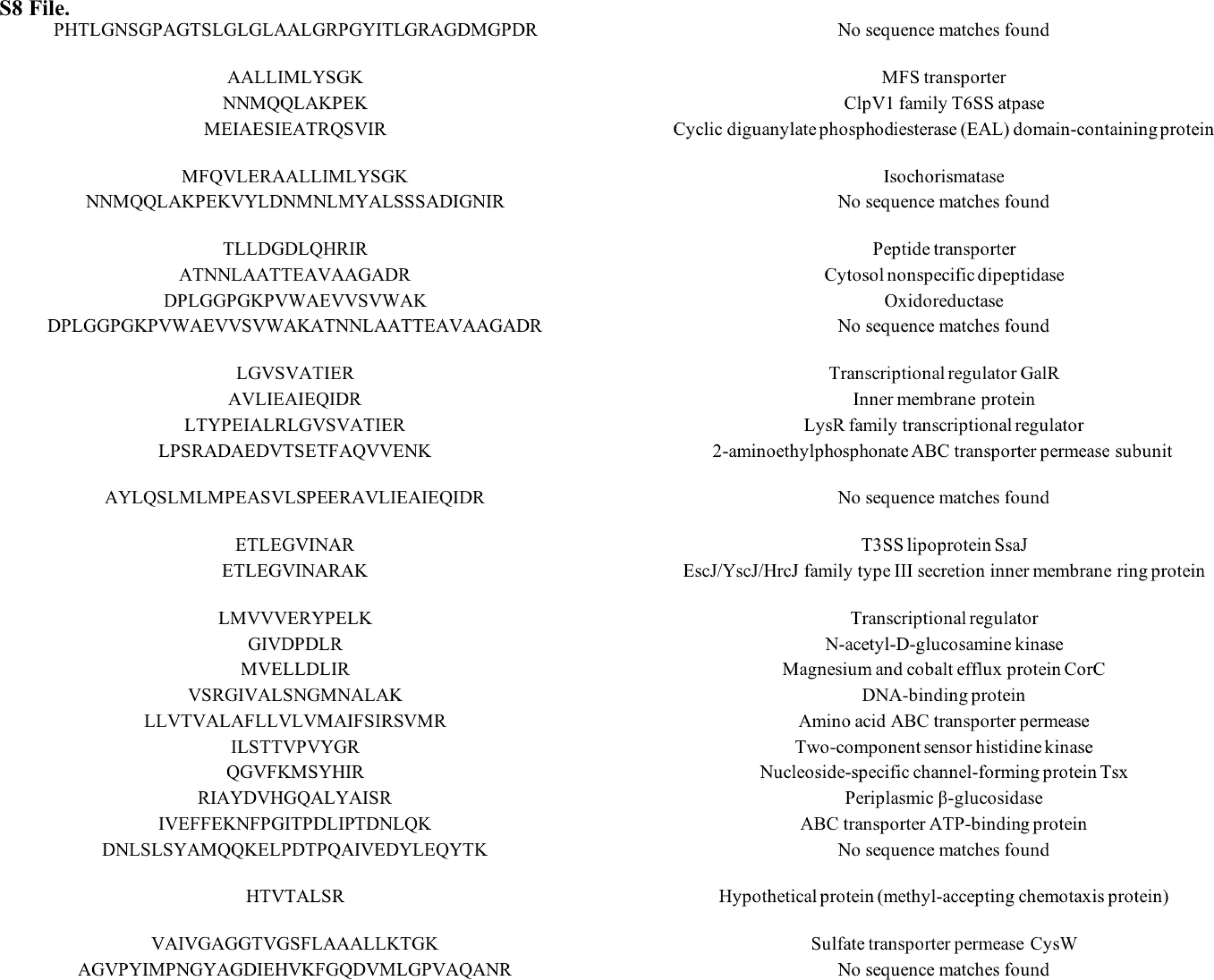

Supplement: S8 File — (TIF) [file pone.0203631.s008.tif]

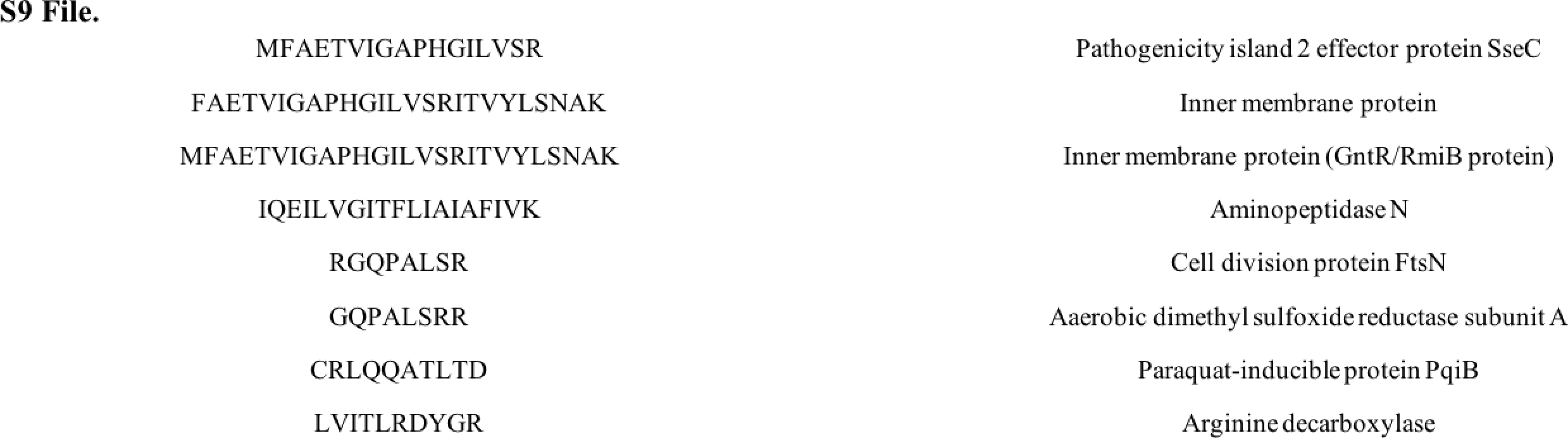

Supplement: S9 File — (TIF) [file pone.0203631.s009.tif]

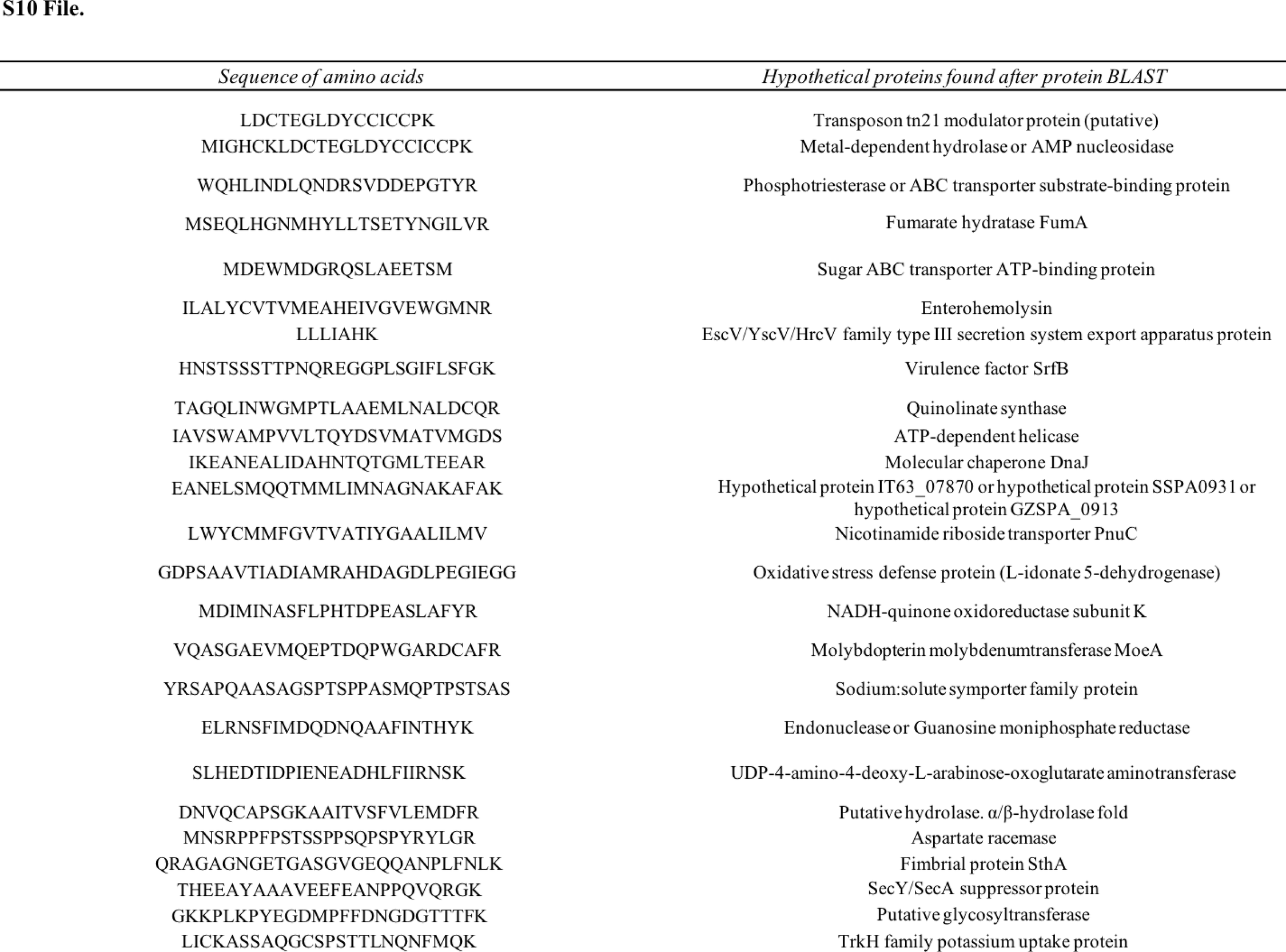

Supplement: S10 File — (TIF) [file pone.0203631.s010.tif]

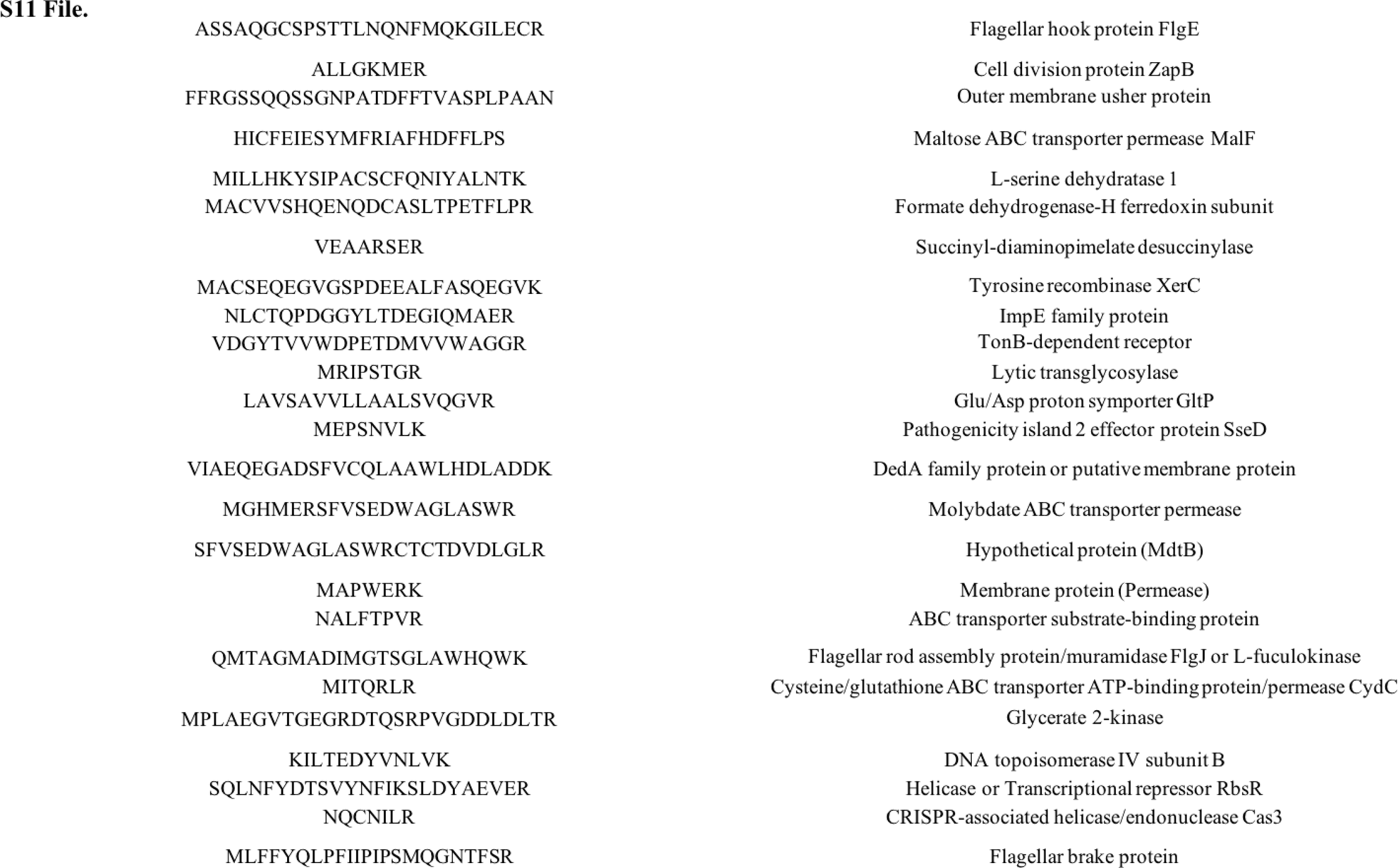

Supplement: S11 File — (TIF) [file pone.0203631.s011.tif]

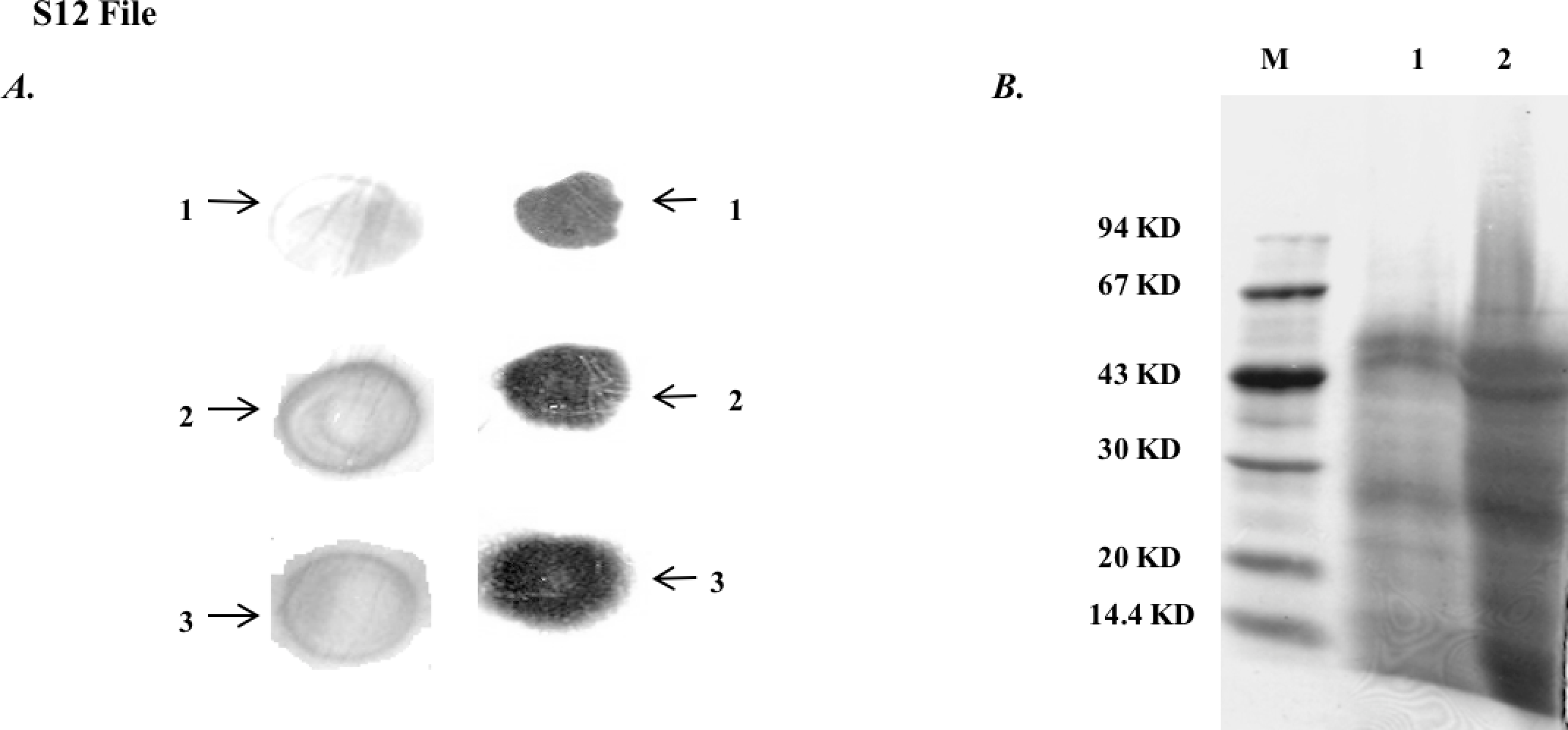

Supplement: S12 File — A. Dot blot analysis against extracted LPS from two typhoidal strains. Lane 1: S. Typhi native LPS, Lane 2: S. Paratyphi A native LPS. Here, 1, 2, and 3 denotes three different concentrations of LPSs against which the dot blot analysis was performed. B.TCA precipitation of culture supernatant. Lane 1: S. Typhi culture supernatant, Lane 2: S. Paratyphi A culture supernatant. (TIF) [file pone.0203631.s012.tif]

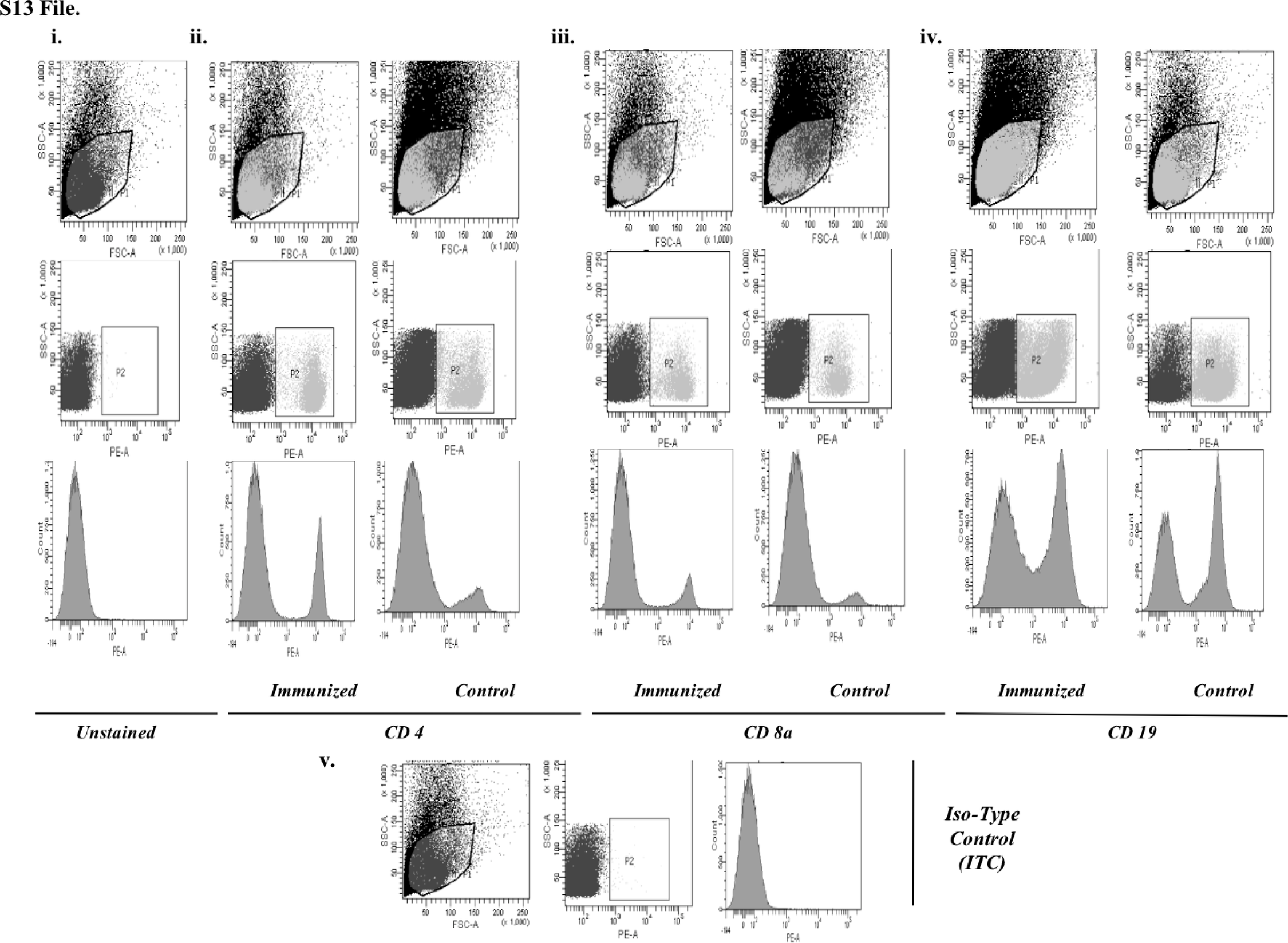

Supplement: S13 File — 50,000 events in total population were taken in each and every case and the mean value of area under PE in P2 population was taken into account whilst calculating the result. i. Unstained cells, ii., iii., iv. Up-regulation of CD4, CD8a and CD19, respectively in immunized and control mice’s spleen. v. REA-cloned Iso-Type Control (ITC) for all three markers. (TIF) [file pone.0203631.s013.tif]

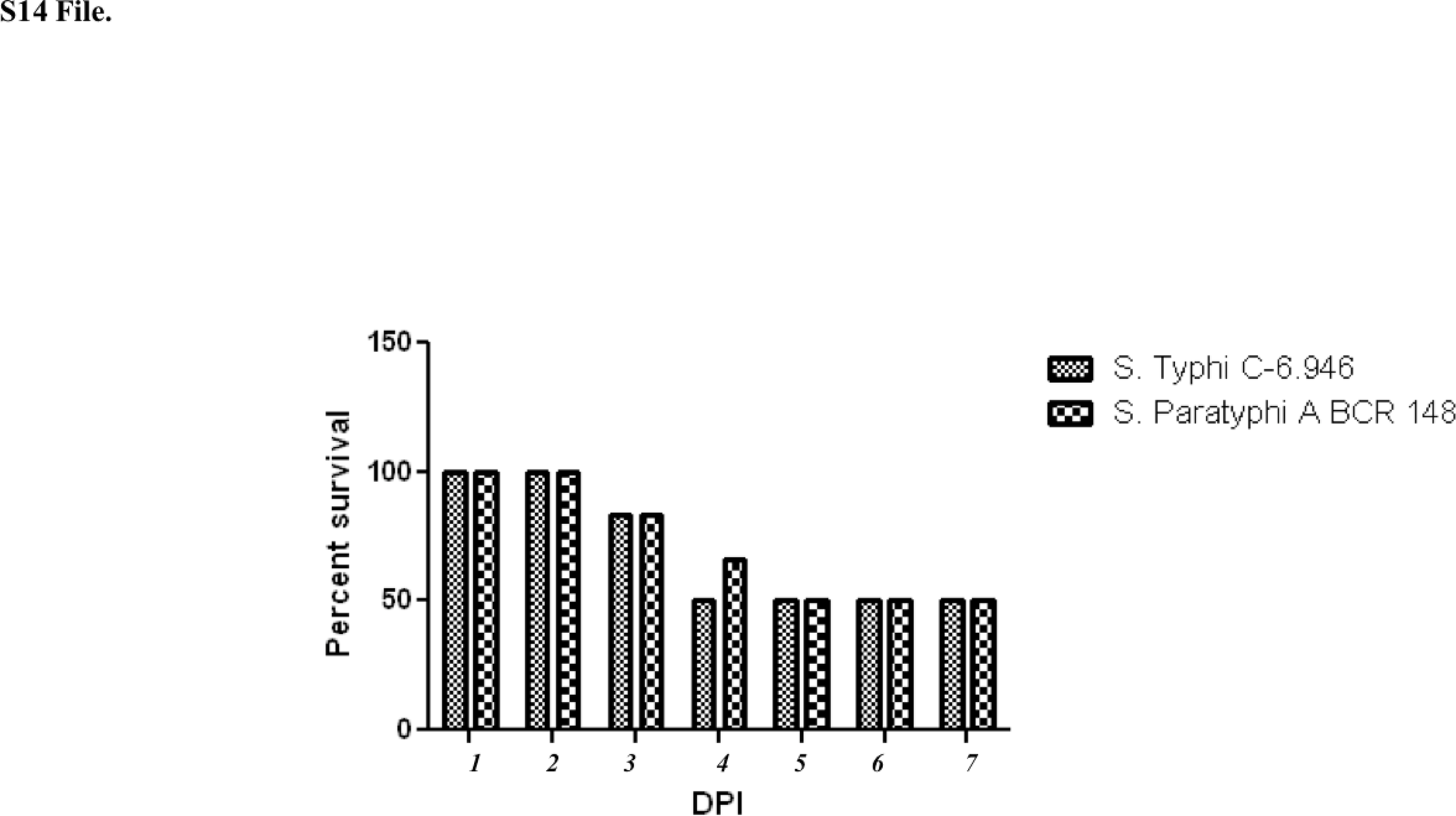

Supplement: S14 File — 6 mice were per group were challenged with different concentrations of bacteria. Mice were observed for 7 days. The effective dose for LD50 or 50% mortality rate was found to be 2 x 10^7^ CFU/ml for both the challenge strains. (TIF) [file pone.0203631.s014.tif]

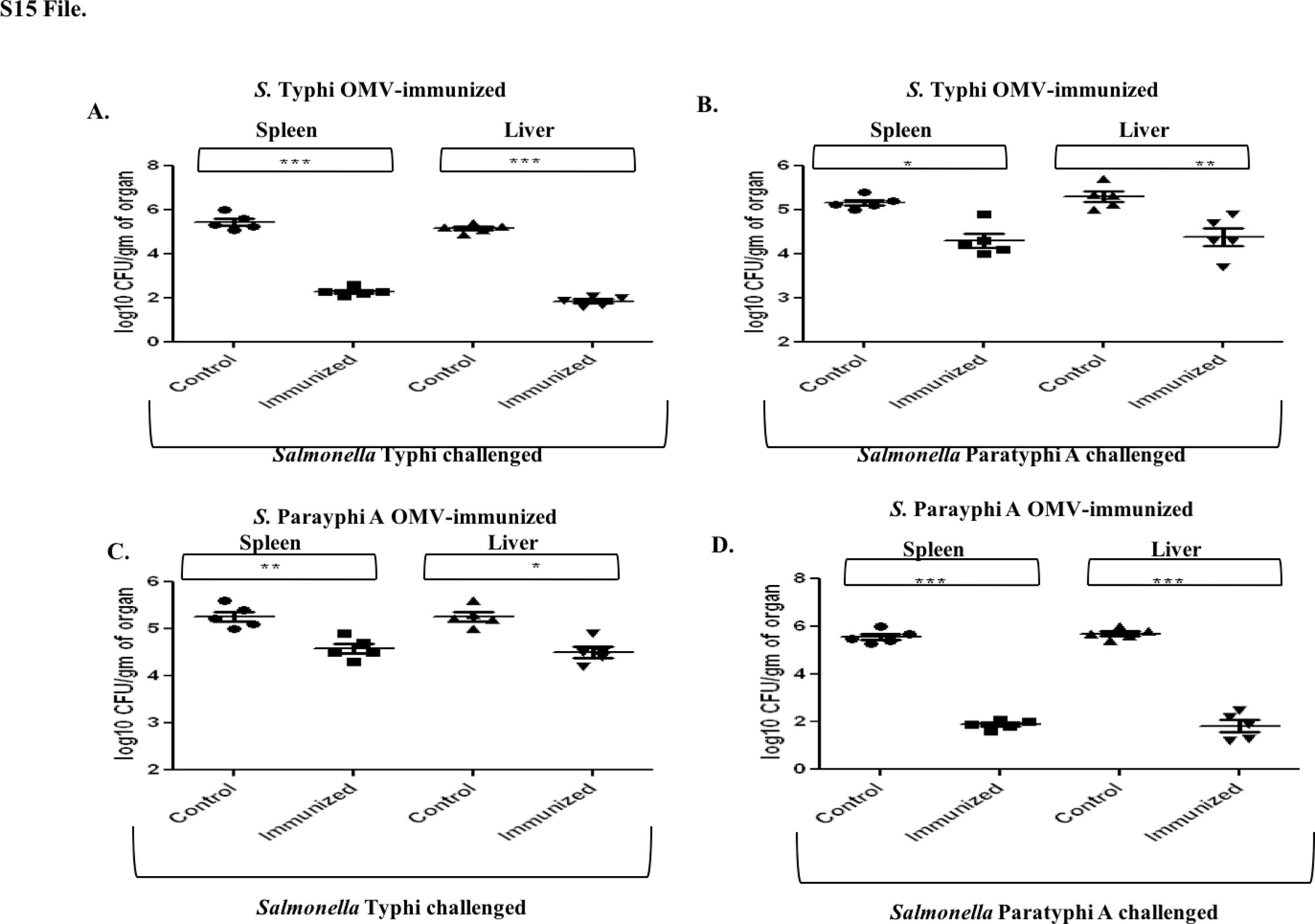

Supplement: S15 File — A., B. Colonization in spleen and liver in S. Typhi OMV-immunized mice were less when they were challenged with S. Typhi. C. D.Colonization in spleen and liver in S. Paratyphi A OMV-immunized mice were less when they were challenged with S. Paratyphi A. In both cases,monovalent OMV immunization could not be able to hinder the infection from a heterologous strain. (TIF) [file pone.0203631.s015.tif]

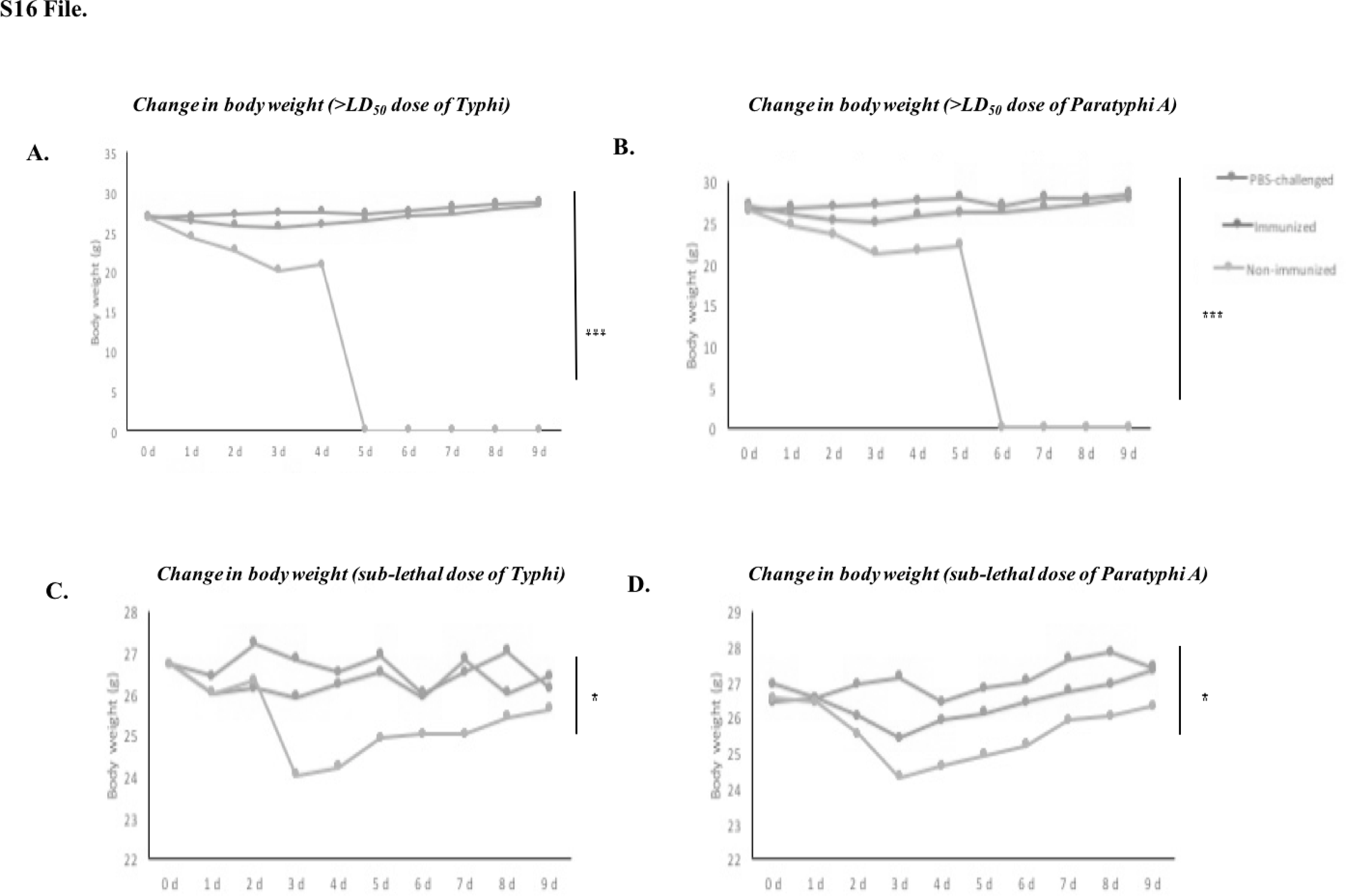

Supplement: S16 File — A., B. A drastic change in body weight was seen in non-immunized mice challenged with >LD50 dose of infection. C. D. A lower level ofchange in body weight was seen in mice challenged with sub-lethal dose of infection. (TIF) [file pone.0203631.s016.tif]

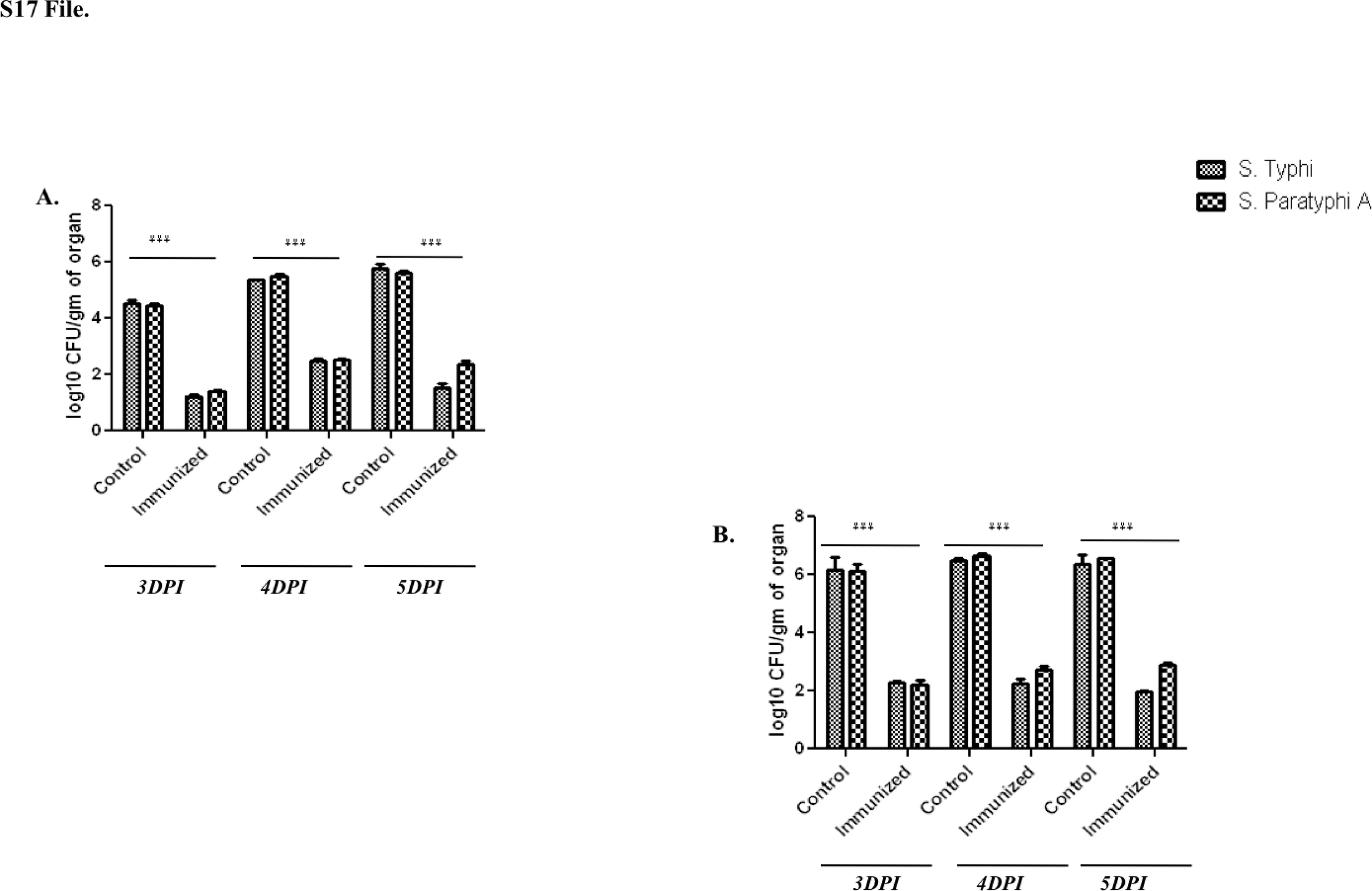

Supplement: S17 File — Three mice per group were challenged either sub-lethal dose or with greater than LD50 dose. Bacterial colonization in the small intestine was estimated since 3DPI until 5DPI. Higher colonization was observed in mice group challenged with greater than LD50 dose. (TIF) [file pone.0203631.s017.tif]

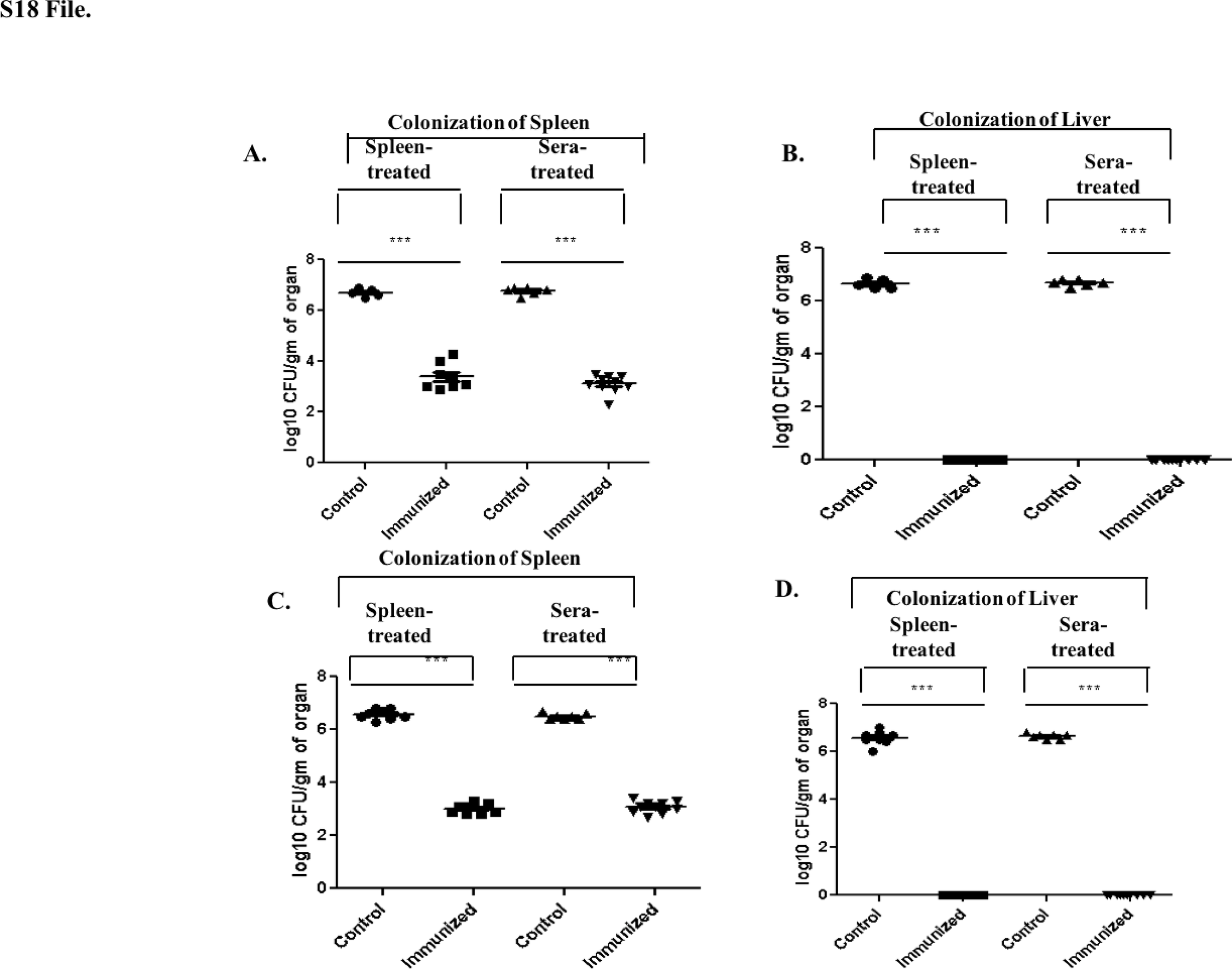

Supplement: S18 File — Both B- and T-cell mediated immune response induced by OMVs is necessary for the protective immunity to bacterial infection. Splenocytes (1 x 10^6^) and serum (100 μl) were injected via the tail vain. One group of mice was challenged with 2 x 10^7^ CFU/ml of heterologous strains of bacteria after two hours of the adoptive transfer and kept for three days. A., B. Systemic infection of S. Typhi in spleen and liver was observed in the early group 3 days post infection. Similarly, C. D. Systemic infection of S. Paratyphi A in spleen and liver was observed 3 days post infection. (TIF) [file pone.0203631.s018.tif]

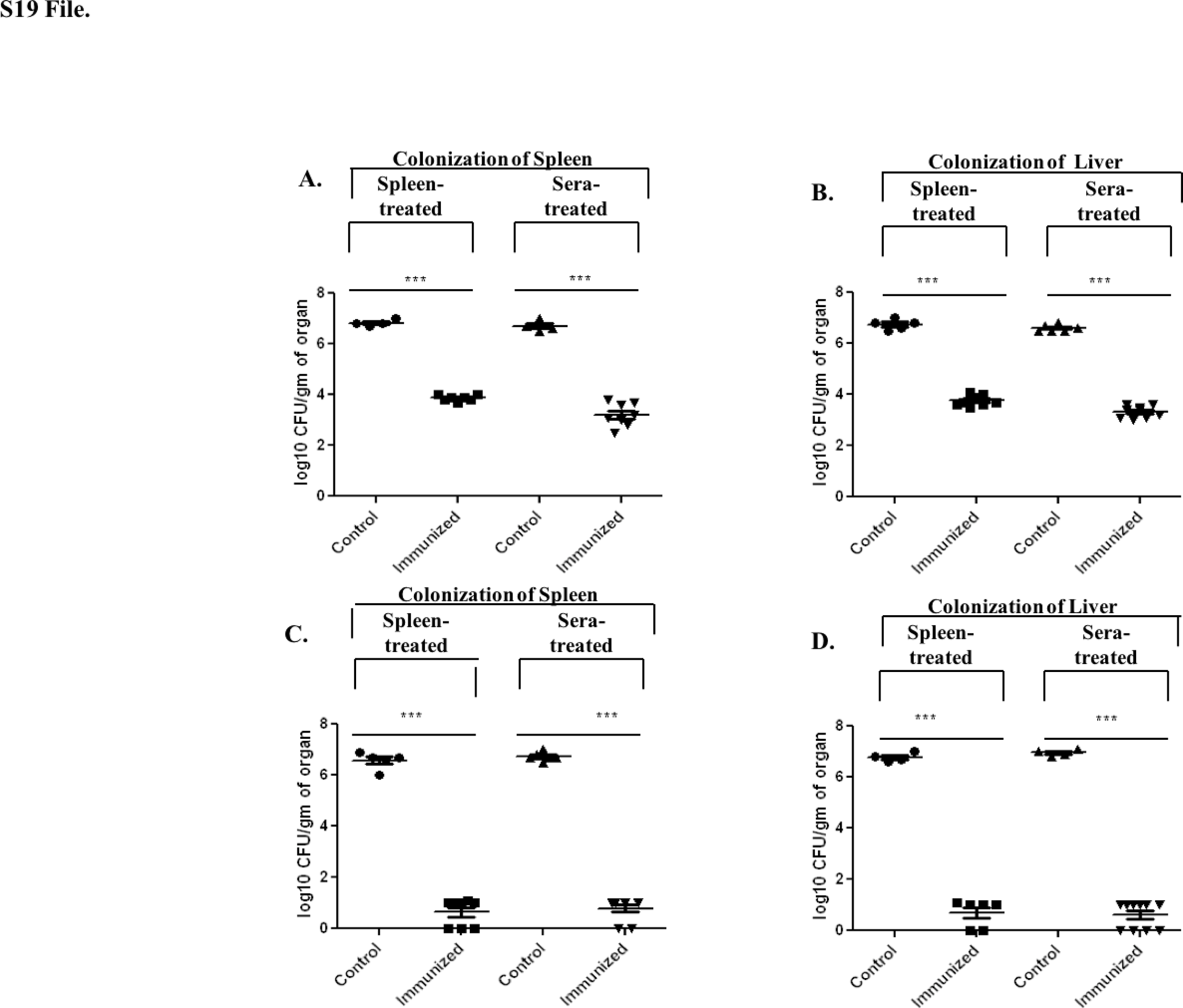

Supplement: S19 File — This group was challenged with the same dose as mentioned above after seven days of the adoptive transfer. A., B. Systemic infection of S. Typhi in spleen and liver wasobserved 3 days post infection. Similarly, C., D. Systemic infection of S. Paratyphi A in spleen and liver was observed 3 days post infection. (TIF) [file pone.0203631.s019.tif]
